# Continuous presence of proto-cereals in Anatolia since 2.3 Ma, and their possible co-evolution with large herbivores and hominins

**DOI:** 10.1038/s41598-021-86423-8

**Published:** 2021-04-26

**Authors:** Valérie Andrieu-Ponel, Pierre Rochette, François Demory, Hülya Alçiçek, Nicolas Boulbes, Didier Bourlès, Cahit Helvacı, Anne-Elisabeth Lebatard, Serdar Mayda, Henri Michaud, Anne-Marie Moigne, Sébastien Nomade, Mireille Perrin, Philippe Ponel, Claire Rambeau, Amélie Vialet, Belinda Gambin, Mehmet Cihat Alçiçek

**Affiliations:** 1 Institut Méditerranéen de Biodiversité et d’Ecologie Marine et Continentale (IMBE), Aix Marseille Univ, Avignon Université, CNRS, IRD, IMBE, Aix-en-Provence, France, Technopôle de l’Environnement Arbois-Méditerranée, BP 80, 13545 Aix-en-Provence Cedex 4, France; 2 Aix-Marseille University, CNRS, IRD, INRAE, UM 34 CEREGE, Technopôle de l’Environnement Arbois-Méditerranée, BP 80, 13545 Aix-en-Provence Cedex 4, France; 3 Department of Geology, Pamukkale University, 20070 Denizli, Turkey; 4 Laboratoire de Préhistoire, Muséum national d’Histoire naturelle, UMR 7194, UPVD, CERP, Avenue Léon Jean Grégory, 66720 Tautavel, France; 5 Department of Geology, Dokuz Eylül University, 35160 İzmir, Turkey; 6 Department of Biology, Ege University, 35100 İzmir, Turkey; 7 Conservatoire Botanique National Méditerranéen de Porquerolles, 34 Av. Gambetta, 83400 Hyères, France; 8 Laboratoire des Sciences du Climat et de l’Environnement (IPSL-CEA-CNRS UMR 8212-UVSQ), CEA Saclay, Site de L’orme Des Merisiers, Bât 714, 91198 Gif Sur Yvette, France; 9 LIVE, UMR7362, Université de Strasbourg, 3 rue de l’Argonne, 67000 Strasbourg, France; 10 Institute of Earth Systems, University of Malta, Msida, Malta

**Keywords:** Palaeontology, Palaeoecology

## Abstract

Cereals are a central resource for the human diet and are traditionally assumed to have evolved from wild grasses at the onset of the Neolithic under the pressure of agriculture. Here we demonstrate that cereals may have a significantly longer and more diverse lineage, based on the study of a 0–2.3 Ma, 601 m long sedimentary core from Lake Acıgöl (South-West Anatolia). Pollen characteristic of cereals is abundant throughout the sedimentary sequence. The presence of large lakes within this arid bioclimatic zone led to the concentration of large herbivore herds, as indicated by the continuous occurrence of coprophilous fungi spores in the record. Our hypothesis is that the effects of overgrazing on soils and herbaceous stratum, during this long period, led to genetic modifications of the Poaceae taxa and to the appearance of proto-cereals. The simultaneous presence of hominins is attested as early as about 1.4 Ma in the lake vicinity, and 1.8 Ma in Georgia and Levant. These ancient hominins probably benefited from the availability of these proto-cereals, rich in nutrients, as well as various other edible plants, opening the way, in this region of the Middle East, to a process of domestication, which reached its full development during the Neolithic.

## Introduction

**Figure 1:**
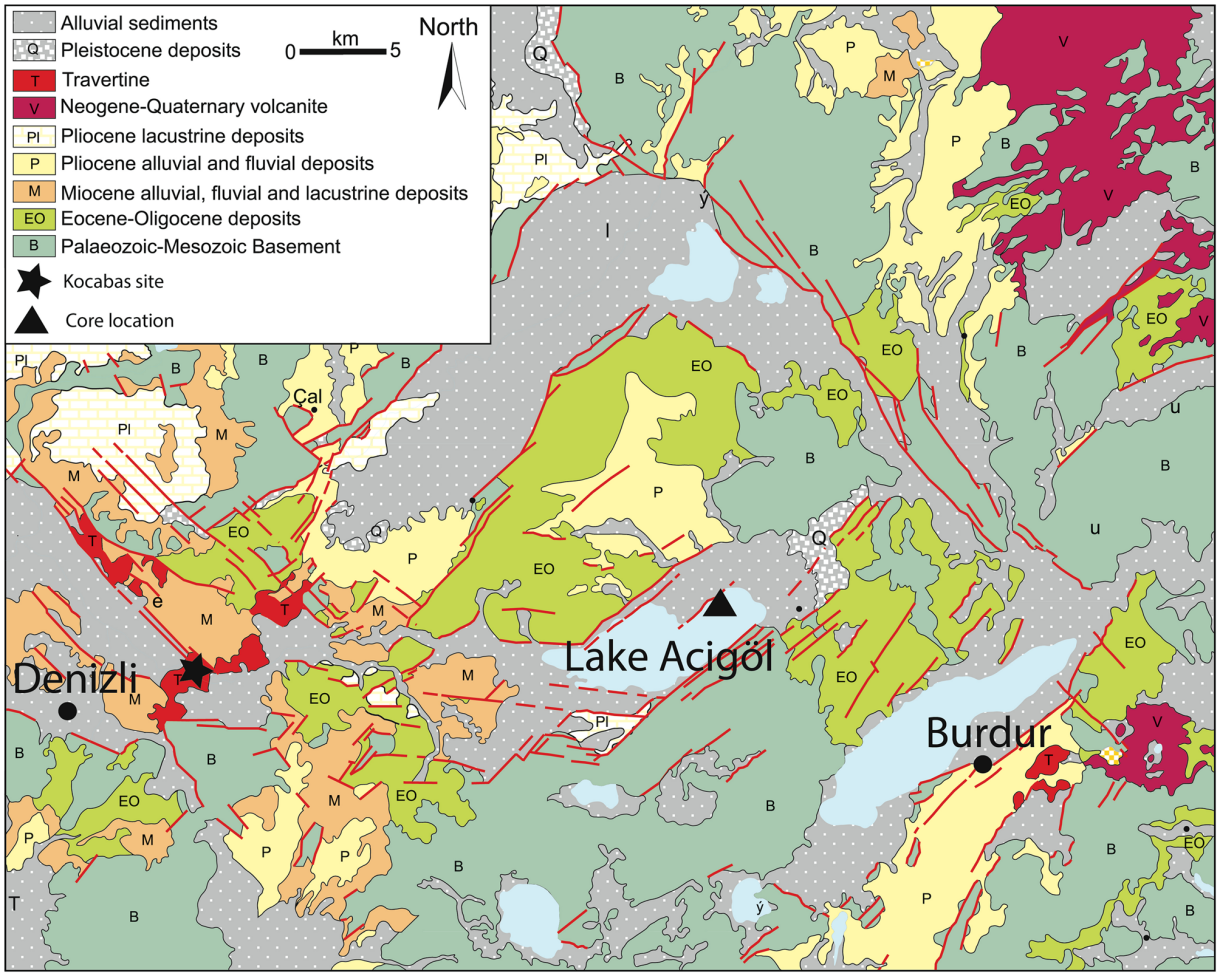
Simplified geological map and location of the sites. Redrawn from Alçiçek et al.^16^

**Figure 2:**
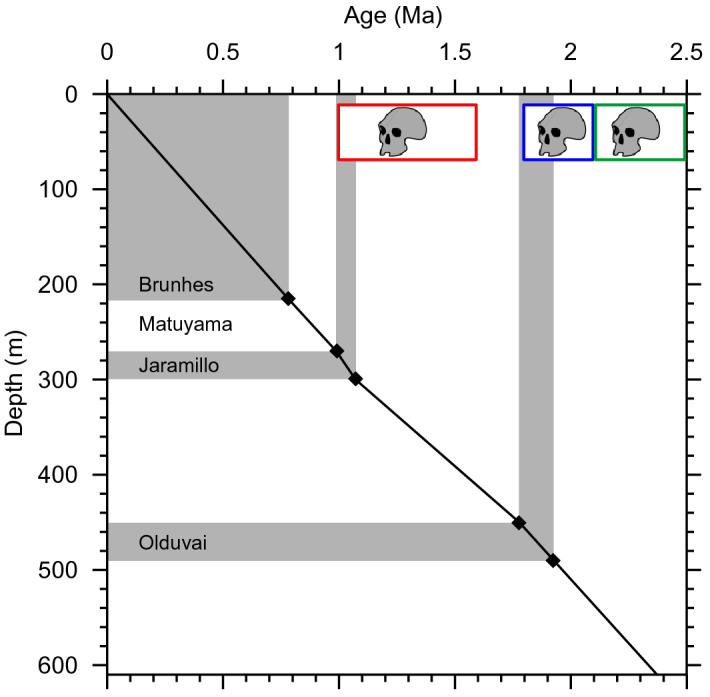
Time/depth curve of Acıgöl core, with paleomagnetic tie point in black, normal geomagnetic polarities in gray. Earliest hominin presence in Anatolia [4], Ciscaucasia [8], China plus Jordan [9, 10], is indicated in red, blue and green, respectively.

An interdisciplinary study was carried out on the lacustrine sedimentary sequence of Acıgöl (Lakes district, SW Turkey, (Fig. 1). Dated from 0 to 2.3 Ma^1^ and extending over 601 m in length, it covers almost the entire Quaternary. With rather constant sedimentation rates, ranging from 21 to 35 cm ka^1^, Lake Acıgöl provides an exceptional palaeoecological archive, and is the longest lacustrine record for western Asia. Age control (Fig. 2) was provided by the paleomagnetic identification of the Bruhnes-Matuyama Boundary, the Jaramillo and Olduvai subchrons, as well as cyclostratigraphy^1^. The glacial/interglacial pseudo-cycles at around 100 ka period were recorded in magnetic susceptibility as well as major elements composition (see methods and^1^). The oldest *Homo erectus* remains from Turkey (the Kocabaş skull) were discovered in the travertine deposit of Denizli 40 km W of Acıgöl lake^2,3^ and dated to ca 1.2–1.6 Ma^4^, documenting one of the main early migratory axis of hominin populations between Africa and Eurasia. Simultaneous or later signs of early Pleistocene human occupation based on lithic tools were found in Anatolia, in particular in the nearby Gediz river site^5^ (120 km NW of Acıgöl) and Dursunlu site (180 km E of Acιgöl). More distant earlier presence of hominins around ~ 1.8 Ma were evidenced in the Levant^6^, Georgia^7^ and Ciscaucasia^8^, pointing toward the high probability of hominin occupation of Anatolia, mid-way of Levant and Caucasus, at least sporadic since 1.8 Ma. Evidence from China and Jordan points toward even earlier presence of hominins out of Africa as early as 2.1^9^ and 2.5 Ma^10^, respectively. In the Denizli and Burdur basins, adjacent to Acıgöl, abundant fossil remains of large mammals typical of the late Villafranchian have been unearthed, including an extinct species of primitive mammoth (*Archidiskodon m. meridionalis*), an extinct rhinoceros (*Stephanorhinus* cf. *etruscus*), several species of horses (*Equus*) sp., *E.* cf. *altidens*/*E.* cf. *mygdoniensis* and *E.* cf. *apolloniensis*), small and large-sized deer (*Metacervoceros* *rhenanus*, *Arvernoceros* sp., *Cervalces (Libralces*) ex gr. *minor-gallicus*), a large primitive okapi (*Palaeotragus* sp.), a primitive camel (*Paracamelus* cf. *gigas*), a large bovine today extinct (*Leptobos* cf. *etruscus*) and several antelopes (*Gazellospira torticornis*, *Gazella* sp.) ^11–14^. They indicate that *Homo erectus* coexisted with a rich and diversified mega-fauna from which they were largely dependent^15^.

## Results

### Vegetation history of the Acıgöl area

**Figure 3:**
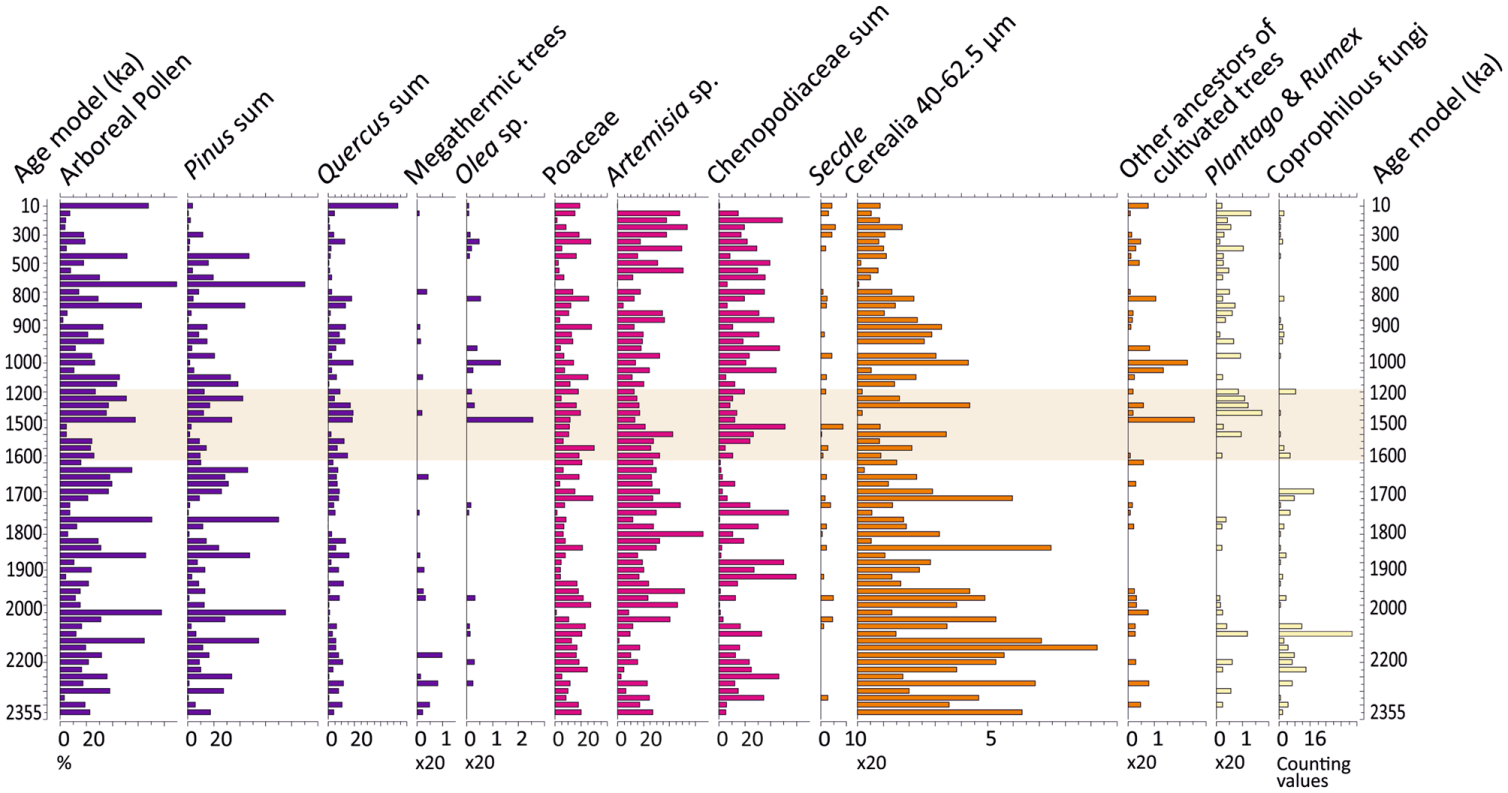
Simplified pollen and NPP diagram in percentages of Acıgöl, core 3, based on the age model of Demory et al. [1]. Equidistant scale. Values are in percentages calculated on a pollen sum without Non-Pollen Palynomorphs (NPP), Ferns, Bryophytes and Algae. The beige rectangle corresponds to the date of the presence of *Homo erectus* at Kocabaş (Lebatard et al. [4]).

Our palynological analyses of 72 regularly spaced samples show a diversified vegetal landscape alternately wooded and open, in response to orbitally driven climatic cyclicity. However, arboreal pollen values remain almost constantly below 50% of the Pollen Sum (PS) (average 27.5%, median 22.8%), which corresponds to an overall open landscape (Fig. 3). Among herbaceous plants, the dominant taxa are steppics such as *Artemisia*, heliophilous and halophilous taxa including *Calystegia*, several Compositae, *Convolvulus*, *Linum*, *Plantago* ssp., Poaceae and Chenopodiaceae that could develop on the saline shores of Acıgöl lake during evaporitic periods. Forests are composed of a mixture of conifers, Mediterranean *Pinus, Abies*, *Cedrus,* Cupressaceae and *Picea,* associated with broadleaved trees dominated by Mediterranean oaks, *i.e.* deciduous and evergreen *Quercus*, with some *Olea*. Riverine trees such as *Alnus, Salix, Populus, Tamarix, Juglans* and *Platanus* have also been identified. Few Tertiary or megathermic relictual taxa (*Carya, Liquidambar, Parrotia*, *Pterocarya fraxinifolia*, Taxodiaceae, *Tsuga, Zelkova*) were identified so far in the pollen assemblages, mostly before 2.2 Ma, due to climatic cooling^17,18^ since the end of Tertiary which led to a decline in global biodiversity^19,20^.

The vast freshwater stretch of Acıgöl, located in a predominantly arid limestone hills environment, seems to have been a crucial resource for the mammalian fauna, which probably concentrated around the site in search of water and pastures. Indeed, low percentages of arboreal pollen imply that the landscape remained open throughout the sequence and suggest a marked grazing pressure by herbivores in addition to climatic factors^21–23^.

### Coprophilous fungi spores, cereals and other ancestors of cultivated plants

**Figure 4:**
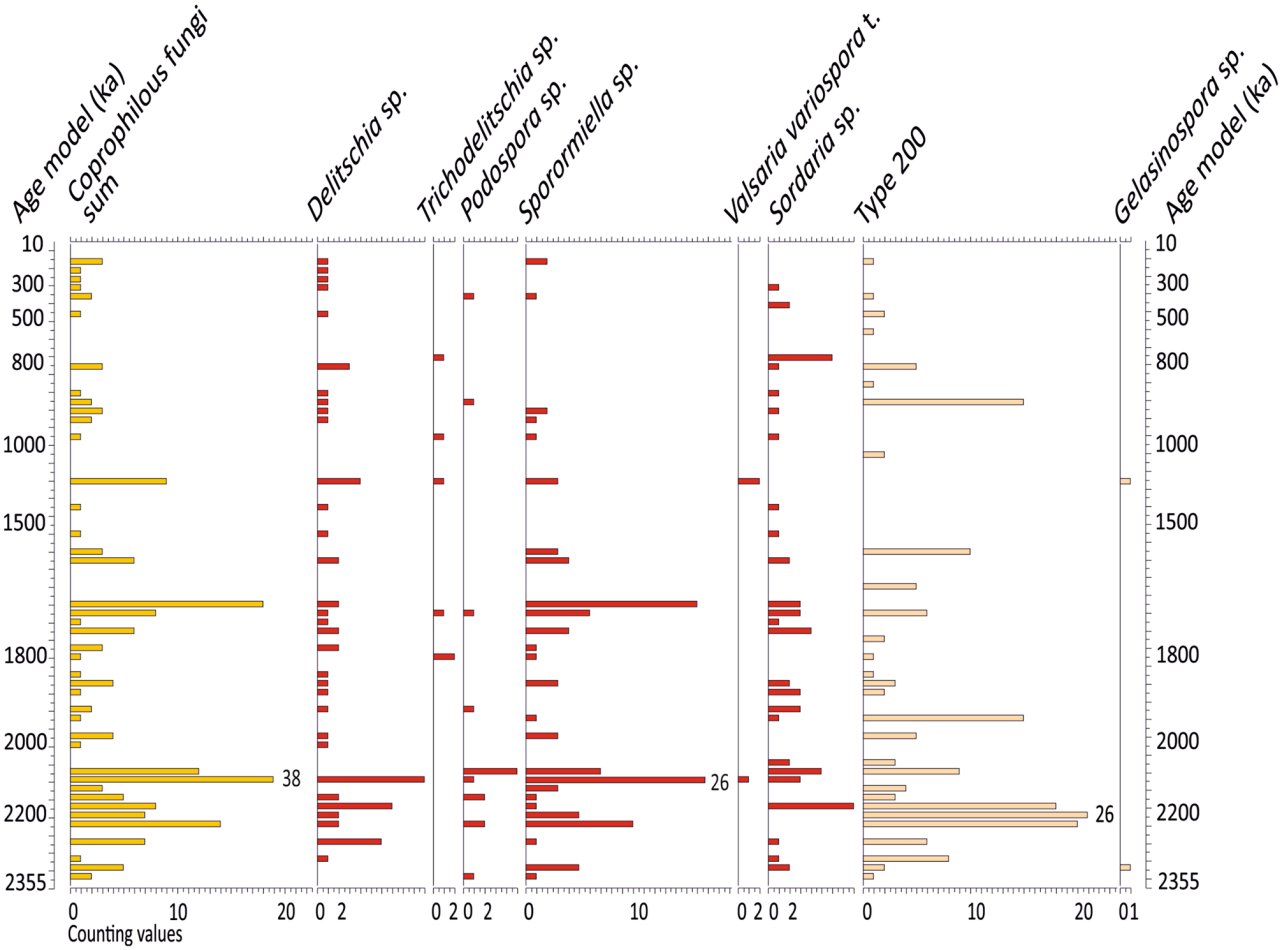
Coprophilous fungi spores of Acıgöl, core 3. Equidistant scale. Age model is from Demory et al. [1]. In red: coprophilous fungi taxa..

Coprophilous fungi spores are excellent indicators of herbivorous mega-mammal herds since they grow exclusively on dung deposited by these animals^24^. At Acıgöl, a wide variety of coprophilous fungi spores has been identified throughout the pollen record including: *Sporormiella* sp*.*, *Podospora* sp., *Delitschia* sp., *Sordaria* sp. and *Valsaria variospora* (Figs. 3, 4). They provide evidence for a continuous presence of large herbivorous mammals around the lake throughout Quaternary.

**Figure 5:**
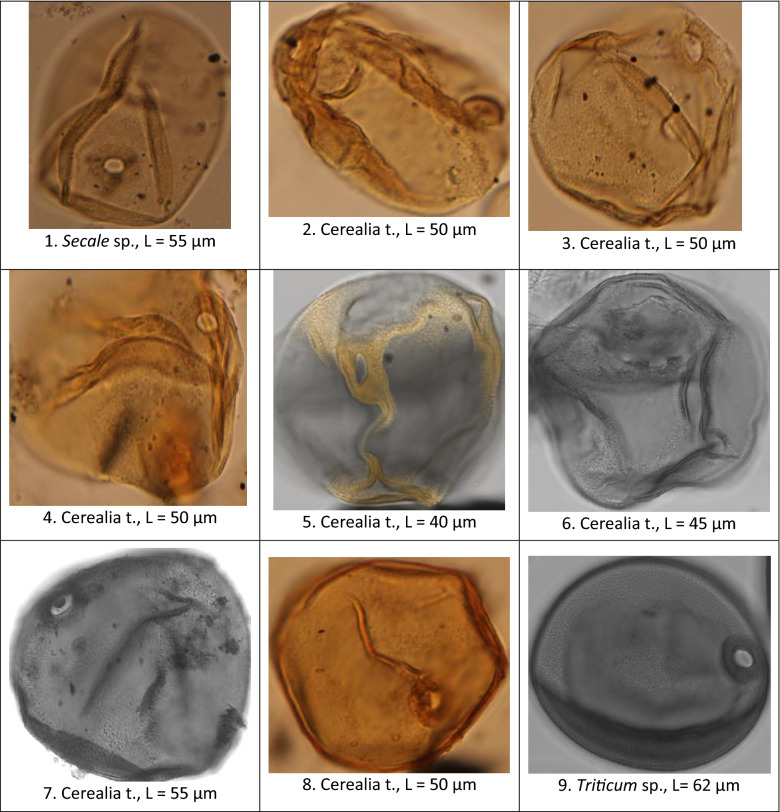
Pollen grain of Cerealia and Triticum sp. from Acıgöl (ACI), core 3 (photos 1–7), the Roman site of La Verrerie, Arles, France (photo 8) and Gardouch, France, current wheat field (photo 9). Photographies with a photonic (photo 1 – 4 and 8) and a confocal microscope (photos 5-7 and 9). 1) sample ACI 239 m, age: 0.871 Ma. 2) sample ACI 435.50 m, age: 1.709 Ma. 3) sample ACI 532.44 m, age: 2.122 Ma. 4) sample ACI 509.50 m, age: 2.026 Ma. 5) sample ACI 552.57 m, age 2.206. 6) sample ACI 552.57 m, age: 2.206 Ma. 7) sample ACI 429.50 m, age: 1.681 Ma. 8) sample La Verrerie 1455, age: 50-70 BC (Roman). 9) current pollen of *Triticum* sp., age: 2000 AD. L: maximal length (µm).

**Figure 6:**
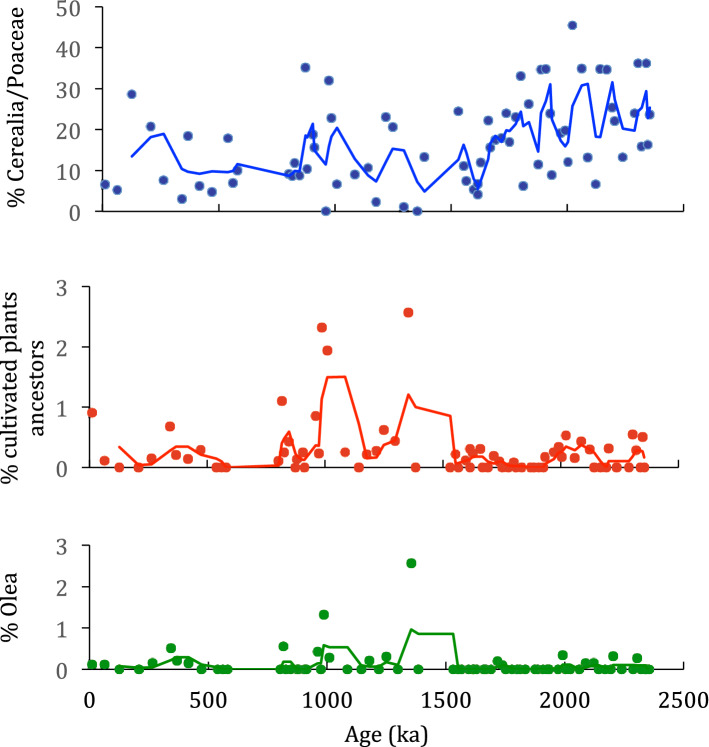
Cerealia/Poaceae ratio in %, % cultivated tree ancestors and % *Olea* of Acıgöl, core 3.

Pollens of Poaceae, such as *Secale* (rye) and Cerealia-type, have been identified throughout the sequence (Figs. 3, 5). Unexpectedly, they present the same morphological characteristics as that of modern cereal grains^25,26^, namely an average size of ≥ 40 µm and a large pore + *annulus* (≥ 8 µm). As by definition cereals are cultivated plants, we will call the corresponding plants “proto-cereals” to highlight that their pollen are identical to those of cereals. This resemblance can be seen clearly in Fig. 5, where we have brought together fossil cereals from Acιgöl (Fig. 5, photos 1–7), from Roman time (Fig. 5, photo 8), not modified by modern agricultural practices, and from the current wheat field of the Lauragais agricultural plain, Gardouch, France (Fig. 5, photo 9). Cerealia-type frequencies reach a maximum of 9% of the PS around 2.2 Ma and can be as abundant as wild Poaceae pollen (Fig. 3). The Cerealia/Poaceae ratio shows that 24.66% of all Poaceae are proto-cereals from 2.0 to 2.3 Ma (Supplementary Table 1). Such high proto-cereal rates are almost never reached in pollen records, even in recent periods and in the presence of agriculture, because of the very low pollen dispersal capacity of cereals^27^. A lowering of frequencies down to 2–4% range is recorded in younger periods (Fig. 3), as well as a step like decrease of the Cerealia/Poaceae ratio (Fig. 6). This change may be related to the Middle-Pleistocene Transition (MPT) cooling and to the mega-mammal fauna change from a Villafranchian to a Galerian type^28^. MPT and faunal changes occurred around 0.9–1.0 Ma, while a decrease in our proto-cereal starts around 1.5 Ma, however signs of cooling and amplified climatic cycles predate the MPT^28^.

**Figure 7:**
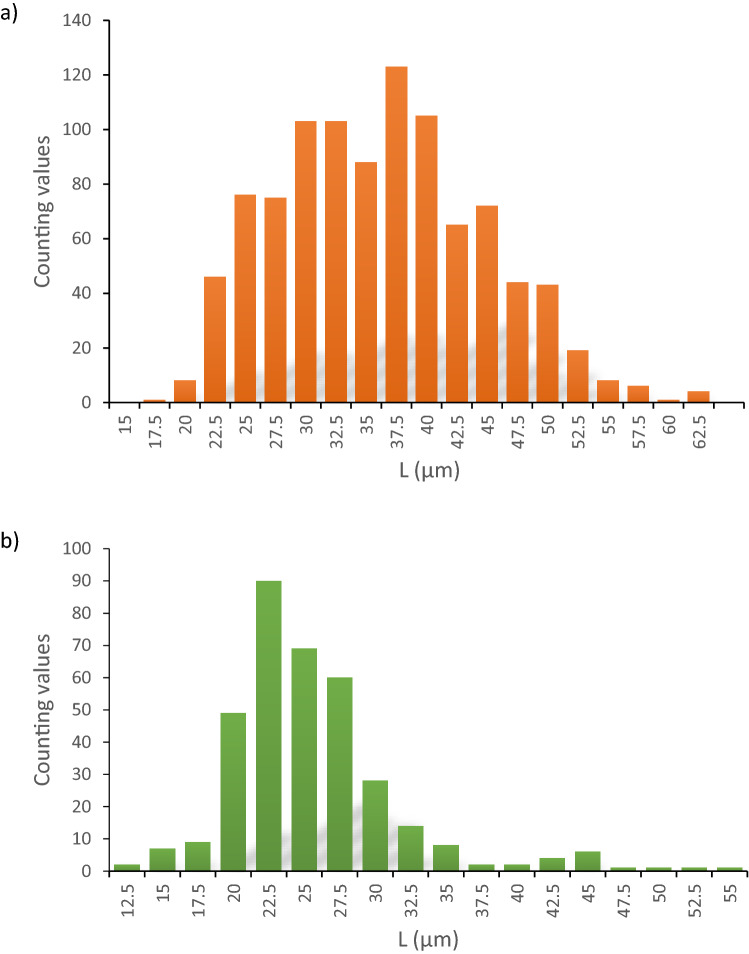
a) Pollen size of wild Poaceae and proto-cereal of Acıgöl, core 3. The measurements were made on the 10 samples with the highest cereal pollen content. A total of 991 grains of pollen were measured. b) Current pollen rain at the Acıgöl lake and surroundings. 8 moss samples were collected and 354 measurements of the longest axis of the wild Poaceae and cereal pollen grain were made.

The histogram of wild Poaceae and proto-cereal pollen size (Fig. 7a) shows that there are a number of pollen populations modes around 30, 37.5, 45–50, supporting the idea that the larger grain sizes cannot be interpreted as a tail of ‘anomalous’ wild Poaceae pollen. Moreover, comparison with the present-day pollen rain recorded in moss pollsters, sampled around the lake of Acıgöl (Fig. 7b and Supplementary Table 2), show that the large pollen size mode (≥ 40 µm) is nowadays nearly absent (0–0.97% of the PS, Cerealia/Poaceae ratio of 4.52%, Supplementary Tables 3 and 4), even in biotopes with wild Poaceae considered to be ancestors of cereals (*Aegilops*, sample 2a, cereal rate: 0.97% of the PS) or with cereals such as *Hordeum* (sample 3a, b and 4, cereal rate 0.31, 0.00, 0.33 of the PS respectively, Supplementary Tables 2 and 3).

Our interpretation is that proto-cereals recorded throughout the Acıgöl sequence derive from wild Poaceae. Their emergence and predominance may have been favoured by the impact of large herbivore herds attracted to Acıgöl lake shores, and through genetic drift. Through the process of trampling, nitrogen enrichment of soils and browsing, large mammal herds could have altered the genotype of proto-cereals naturally present in Acıgöl and thus, favoured the emergence of modern cereals. For genetic reasons, the descendants of these proto-cereals are not represented today among cultivated Poaceae because domestication bottlenecks eliminate genetic variation^29^.

Is there a relationship between the size of proto-cereal pollen and climate? To our knowledge, the genetic literature does not show any relationship between the increase in pollen size and temperature. However, there does seem to be a relationship with atmospheric drought^30,31^ which is said to have favoured the appearance of polyploidy in certain species of Poaceae. It cannot be excluded that climate has had an influence on the proto-cereal genome, but only the interaction between herds of large herbivores and proto-cereal steppes can explain why proto-cereal pollen has never been found in such abundance elsewhere in Pleistocene pollen records.

The ancestors of cultivated trees (*Olea* sp., *Juglans* sp., *Castanea* sp., *Corylus* sp., *Prunus* t.), typical of the modern Mediterranean agriculture, are also present in the Acıgöl sequence (Fig. 3 and Supplementary Table 5). Their amount increases after 1.5 Ma, mainly due to *Olea* (Fig. 6). Other potentially edible plants such as *Ephedra*, *Hippophae*, all the Compositae and the Fagaceae have been identified in the pollen assemblages. They correspond to 54.4% of plants identified in the pollen assemblages. Among these plants, there are 72% grasses and 28% trees and, among edible organs, 51% are vegetables and 20% are seeds (Supplementary Fig. 1a,b). These results testify to the potential wealth of accessible food resources that human and animal populations could feed on. Interestingly, studies carried out in Spain on the present-day consumption of wild plants lead to results close to those obtained at Acıgöl, with 87% grasses and 13% trees^32^.

In recent years, new biological and archaeological data obtained from sites with human occupation have improved our knowledge of the beginnings of agriculture and the modalities of its implementation. In the Levant, the Ohalo II site highlights the presence of proto-cereal seeds, and flint tools to harvest, as early as 23,000 years before the present^33^. Further north, on the archaeological site of Gesher Benot Ya'aqov, proto-cereal seeds (oats, *Avena*) as well as pollen from cereals and trees currently cultivated, were identified over a period ranging from 750,000 to 820,000 years^34,35^. Moreover, recent genetic data indicate that the emergence of agriculture did not occur at a single location at the onset of the Neolithic (*e.g.* the "Fertile Crescent" hypothesis) but is, on the contrary, an evolutionary and multi-regional long-term phenomenon^36–38^. Alternatively, or simultaneously, are the hominins also partly responsible by having developed episodes of a form of transitory "proto-agriculture"? We already know that this domestication process was discontinuous with shutdown and restart phases^37,39^. Acheulean lithic tools, characterised by symmetrically shaped bifaces, testify to the rather advanced cognitive capacities of early Pleistocene populations that may have visited the lakeshore of Acıgöl^5^. Hominin populations may also have benefited from this opportunity to diversify their food regime with easily harvested and nutrient-rich wild plants (Supplementary Table 5), as it is the case today for hunter-gatherer populations in Africa and elsewhere in the world.

## Discussion

### Challenging the paradigm of domestication: how and when?

The question surrounding the Neolithic emergence with plant and animal domestication has been debated for decades and has been the subject of countless studies, in most cases carried out by archaeologists and geneticists focusing either on plant macrorests from archaeological sites or on the genome history of cultivated plants^40,41^. The study of natural environments (wetlands, lakes), such as at Acıgöl, and of their microbiological content has so far been largely neglected to tackle this question.

**Figure 8:**
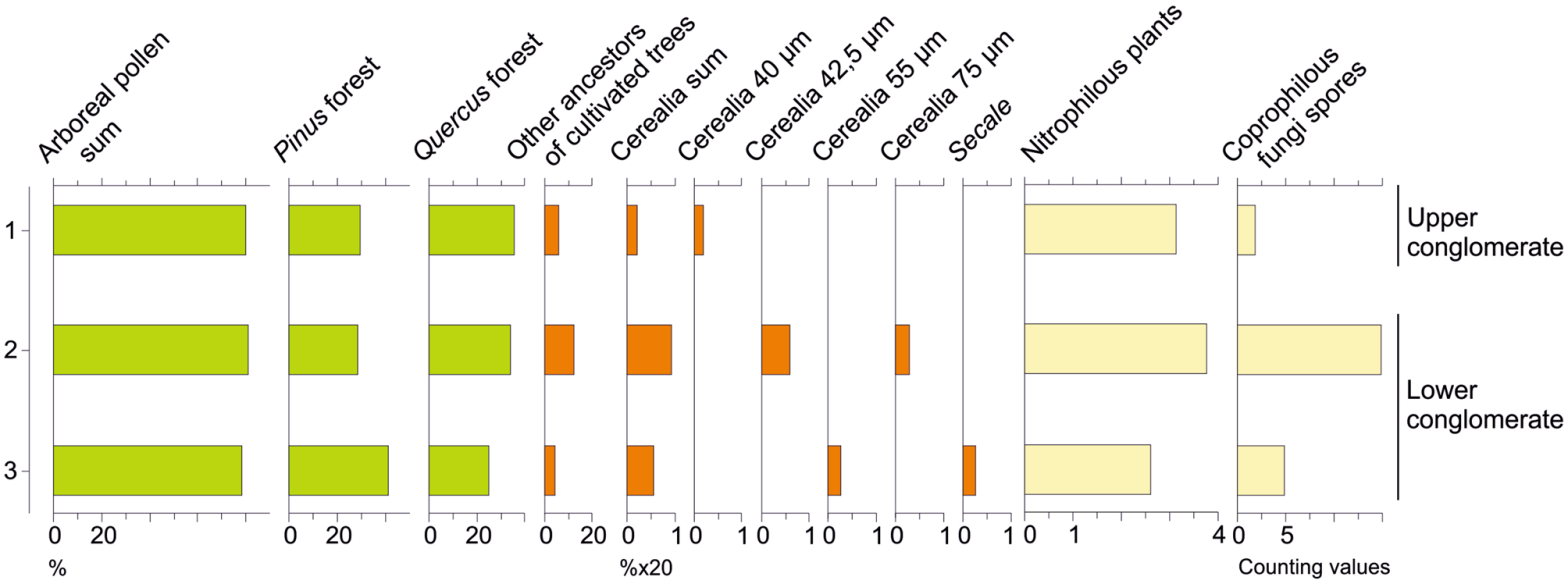
Simplified pollen and NPP diagram in percentages of Kocabaş (Faber quarry, 37°51’N, 29°21’E., 580 m alt.). 3 samples were taken in the fluviatile facies (lower and upper conglomerate) of the travertine formation (details in Lebatard et al. [4]).

What happened in the Neolithic, when humans went from a hunter-gatherer to a farmer lifestyle? Did they reproduce conditions that existed two million years ago? Has there been a new stage of cereal speciation linked to humans? Vaughan^42^ emphasises that "the time scale of domestication of 10,000 years or less is a very short evolutionary time span" (p. 893). The proto-cereal pollen of Acıgöl appears to indicate that the genetic modification of cereals could have also been a natural process that appeared long before agriculture emerged, and that the conditions were already present when human populations shifted from hunter-gatherer to agricultural societies. Our results enable an important enigma to be reformulated in relation to human evolution: when did cereals appear and are humans solely responsible? Our study appears to challenge the long-held paradigm that humans were the progenitors of cereal grasses, when it seems in fact possible that cereal grasses may have appeared naturally, with humans simply accelerating their expansion. If this were confirmed by the presence of proto-cereal pollen in sediments of the lower Pleistocene or in older sediments from other regions, this would necessitate a fundamental revision of our overall vision of the history of human nutrition. To substantiate this, we can report our initial observation of similar proto-cereals in the Kocabaş travertine sequence (Fig. 8) at the early *Homo erectus* stage (1.2–1.6 Ma).

## Material and methods

### Chronostratigraphy

188 oriented standard cylinders were regularly subsampled in Acıgöl core C3, from 100 to 600 m depth. Natural remanent magnetization (NRM) were measured with the Superconducting Rock Magnetometer (SRM 560R, 2G Enterprises) of the Rock Magnetic Laboratory in CEREGE. Demagnetization was mostly performed using alternating magnetic fields. Magnetostratigraphy^1^ allowed identifying the temporal tie points listed in Supplementary Table 6. Bruhnes age cyclostratigraphy based of Xray fluorescence analyses was proposed by Akcer-On^43^, leading to an estimated age of 700 ka at 170 m, coherent with the magnetostratigraphy. 566 samples were used to build a magnetic susceptibility curve versus depth, pointing toward a regular climatic cyclicity record throughout the core, in agreement with sedimentological and palynological proxies^1^.

### Fossil pollen

Pollen analyses were conducted on 87 samples, of which 72 were polliniferous. Non-polliniferous samples correspond to micro-tephra layers (markers of volcanic eruptions or redeposition of detrital tephra) or levels rich in microcharcoals (fire indicators) or algae (blooms of *Botryococcus* or Chrysophyceae in response to a disturbance of the lacustrine trophic system). At Kocabaş, pollen analyses were conducted on three samples taken from fluviatile conglomerates, on both sides of the travertine deposit in which the remains of the Kocabaş Man were discovered and dated to 1.2–1.6 Ma^4^.

The sporo-pollinic material was extracted from the sediment using chemical treatment (HCl acid, NaOH 10% and acetolysis) followed by flotation in heavy liquid (d = 2) and 160 + 10 µm sievings^44^. The identification of pollen, spores and Non-Pollen Palynomorph (NPP) was carried out with a photonic microscope Biomed Leitz (500× magnification). Standard palynological identifications were based on the pollen reference collection of IMBE (CNRS, Aix-en-Provence, France), pollen photographic atlases^45–48^ and articles on NPP^49–51^. The pollen percentages were calculated on a Pollen Sum (PS) including all plants except Filicophytes, Bryophytes, Algae, and NPP. For each sample, pollen counting was carried out up to 300 to 400 grains (phanerogams alone) and then continued over the whole slide for rare taxa^52^. At Acıgöl, the mean PS varies from 3025 grains (all pollen, spores and NPP included) to 558 grains per sample (all pollen, without spores and NPP). The average pollen concentration (weighting method^53^) is of 7829 pollen/g sediment (minimum: 38, maximum: 451,556). The average weight of the samples is 11.24 g (minimum: 3.5 g, maximum: 16.6 g). The total number of taxa identified (pollen, spores, NPP, algae) is 201. At Kocabaş, the mean PS varies from 356 grains (all pollen, spores and NPP included) to 314 grains per sample (all pollen without spores and NPP). The average pollen concentration is 6.5 pollen/g sediment (minimum: 2.9, maximum: 9.9). The weight of the samples is 250 g each. The total number of taxa identified (pollen, spores, NPP, algae) is 69.

The identification of pollen of cereals relies on morphological characteristics^25,53^, especially the longest axis of the grain which is a reliable means to discriminate cereal grains, and the pore + *annulus* diameter which can be used as a complementary criterion. According to Andersen^25^, pollen of Poaceae with a longest diameter larger than 37 µm are pollen of cereals. For Bastin^54^ and Leroi-Gourhan^55^, the diameter of cereals should be ≥ 40 µm and the external diameter of the *annulus* should be at least 10 µm. Detailed information can be found in Emery-Barbier and Thiébault^56^. In our work, we have followed the proposal of Bastin^54^ and Leroi-Gourhan^55^ and designated as cereals the pollen of Poaceae with (1) a longest diameter ≥ 40 µm, (2) a large and protuberant *annulus* + pore, (3) and scabrate to verrucate surface sculpturing of the exine. We thus call proto-cereals (or Cerealia type) the Poaceae pollen ≥ 40 µm and wild Poaceae the others, although we acknowledge that some rare wild Poaceae may show diameter ≥ 40 µm, such as *Aegylops* sp., *Glyceria* sp.^57^. The measurements made on 991 pollen grain of wild Poaceae and proto-cereals of 10 samples of Acıgöl (core 3) chosen among the richest in pollen of cereals (Supplementary Table 1) show that the distribution is polymodal (Supplementary Fig. 1a). The mean pollen diameter of wild Poaceae and cereal is respectively 31.01 and 45.07 µm. The mean pore diameter is 4.28 µm (minimum: 2.5 µm; maximum: 5 µm) and the mean pore + *annulus* diameter is 10.12 µm (minimum: 6.25 µm; maximum: 13.75 µm). In the literature, the mean size of the cereal pore is 2.7 µm^57^. There is no relationship between the diameter of the wild Poaceae and proto-cereal and the size of the pore (R^2^ = 0.046) or the pore + *annulus* (R^2^ = 0.097).

### Modern pollen rain

Sampling of moss pollsters were carried out in May 2017 along the Acıgöl lakeshores and surroundings, in a wide variety of vegetal landscapes, altitudes, and anthropisation levels (Supplementary Table 2), in order to reconstruct the modern pollen rain and to improve the accuracy of palaeoenvironmental reconstructions based on fossil pollen analyses. In this article, we show the results of the pollen analysis of  eight samples. The samples were taken on the lakeshores, in specific ecosystems with halophytic or unsalted vegetation. The objective was to evaluate the amount of cereals in the current pollen rain and to find out whether or not cereal pollen found in Acıgöl sediments may have originated from the hygrophilous vegetation of the lake shoreline. The results show that the pollen of cereal is underrepresented in the current pollen rain of Acıgöl (range of values between 0 and 0.97% of the PS, Cerealia/Poaceae ratio 4.52% against 16.9% (n = 72) in the Acıgöl core Supplementary Tables 3 and 4). This indicates that there is not a biological specificity among the Poaceae community of the Acıgöl lakeshore and that there is a good chance that the proto-cereal pollen of the Acıgöl sedimentary archives come from the surrounding steppe communities and not from hygrophilous wild Poaceae.

## Supplementary Information

Supplementary Information.

Supplementary Figure 1.

Supplementary Table 1.

Supplementary Table 2.

Supplementary Table 3.

Supplementary Table 4.

Supplementary Table 5.

Supplementary Table 6.

## Data Availability

All data generated or analysed during this study are included in this published article (and its Supplementary Information files) and are available in zenodo at https://zenodo.org/record/5912616.

## References

[1] Demory F (2020). Chronostratigraphy, depositional patterns and climatic imprints in Lake Acıgöl (SW Anatolia) during the Quaternary. Quatern. Geochronol..

[2] Kappelman J (2008). First *Homo erectus* from Turkey and implications for migrations into temperate Eurasia. Am. J. Phys. Anthropol..

[3] Vialet, A. et al. The Kocabaş hominin (Denizli Basin, Turkey) at the crossroads of Eurasia. New insight from morphometrical and cladistical analyses. C.R. Palevol., 17, 17-32 (2018).

[4] Lebatard, A.E. *et al.* Dating the *Homo erectus* bearing travertine from Kocabaş (Denizli, Turkey) at least 1.1 Ma. *Earth Planet. Sci. Lett.* **390**, 8–18 (2014).

[5] Maddy D (2015). The earliest securely-dated hominin artefact in Anatolia?. Quatern. Sci. Rev..

[6] Ron H, Levi S (2001). When did hominids first leave Africa? New high-resolution magnetostratigraphy from the Erk-el-Ahmar Formation, Israel. Geology.

[7] Vekua A (2002). A new skull of early *Homo* from Dmanisi, Georgia. Science.

[8] Shchelinsky VE (2016). The Early Pleistocene site of Kermek in western Ciscaucasia (southern Russia): Stratigraphy, biotic record and lithic industry (preliminary results). Quatern. Int..

[9] Zhu, Z., Dennell, R., Huang, W. *et al.* Hominin occupation of the Chinese Loess Plateau since about 2.1 million years ago. *Nature* **559,** 608–612 (2018). 10.1038/s41586-018-0299-4 29995848

[10] Scardia, G. *et al.* Chronologic constraints on hominin dispersal outside Africa since 2.48 Ma from the Zarqa Valley, Jordan. *Quatern. Sci. Rev.* **219**, 1–19 (2019).

[11] Alçiçek MC (2013). Lower Pleistocene stratigraphy of the Burdur Basin of SW Anatolia. C.R. Palevol.

[12] Boulbes N (2014). Les grands mammifères du Villafranchien supérieur des travertins du Bassin de Denizli (Sud-Ouest Anatolie, Turquie). Anthropologie.

[13] Demirel FA, Mayda S (2014). A new Early Pleistocene mammalian fauna from Burdur Basin, SW Turkey. Russ. J. Theriol..

[14] Rausch Lea, Alçiçek Hülya, Vialet Amélie, Boulbes Nicolas, Mayda Serdar, Titov Vadim V., Stoica Marius, Charbonnier Sylvain, Abels Hemmo A., Tesakov Alexey S., Moigne Anne-Marie, Andrieu-Ponel Valerie, De Franceschi Dario, Neubauer Thomas A., Wesselingh Frank P., Cihat Alçiçek M. (2019). An integrated reconstruction of the early Pleistocene palaeoenvironment of *Homo erectus* in the Denizli Basin (SW Turkey). Geobios.

[15] Agusti, J. & Anton, M. *Mammoths, Sabertooths, and Hominids. 65 Million Years of Mammalian Evolution in Europe.* (Columbia Univ. Press, 2000).

[16] Alçiçek MC (2013). Superimposed basin formation during Neogene-Quaternary extensional tectonics inSW-Anatolia (Turkey): Insights from the kinematics of theDinar Fault Zone. Tectonophysics.

[17] Lisiecki LE, Raymo ME (2005). A Plio-Pleistocene stack of 57 globally distributed benthic δ18O records. Paleoceanography.

[18] Herman F, Champagnac JD (2016). Plio-Pleistocene increase of erosion rates in mountain belts in response to climate change. Terra Nova.

[19] Biltekin D (2015). Anatolia: A long-time plant refuge area documented by pollen records over the last 23 million years. Rev. Palaeobot. Palynol..

[20] Combourieu Nebout, N. & Vergnaud Grazzini, C. Late Pliocene northern hemisphere glaciations: The continental and marine responses in the central Mediterranean. *Quatern. Sci. Rev.* **10**, 319–334 (1991).

[21] Olff H, Ritchie ME (1998). Effects of herbivores on grassland plant diversity. Trends Ecol. Evol..

[22] Augustine, D.J. & Mc Naughton, S.J. Regulation of shrub dynamics by native browsing ungulates on East African rangeland. *J. Appl. Ecol.* **41**, 45–58 (2004).

[23] Githumbi EN (2018). Pollen, people and place: Multidisciplinary perspectives on ecosystem change at Amboseli, Kenya. Front. Earth Sci..

[24] Baker AG, Bhagwat SA, Willis KJ (2013). Do dung fungal spores make a good proxy for past distribution of large herbivores?. Quatern. Sci. Rev..

[25] Andersen, S.T. Identification of wild grass and cereal pollen. *Danm. Geol. Underst*. 69–92 (1979).

[26] Faegri, K. and Iversen, J. *Textbook of Pollen Analysis*. 4th edn (revised by Faegri, K., Kaland, P.E. & Krzywinski, K.). (Wiley, Chichester, 1989).

[27] Vuorela I (1973). Relative pollen rain around cultivated fields. Acta Bot. Fenn..

[28] Muttoni G (2010). Human migration into Europe during the late Early Pleistocene climate transition. Palaeogeogr. Palaeoclimatol. Palaeoecol..

[29] Olsen KM, Gross BL (2008). Detecting multiple origins of domesticated crops. PNAS.

[30] Manzaneda AJ (2012). Environmental aridity is associated with cytotype segregation and polyploidy occurrence in *Brachypodium distachyon* (Poaceae). New Phytol..

[31] Ejsmond, M.J. *et al.*, Does climate affect pollen morphology? Optimal size and shape of pollen grains under various desiccation intensity. *Ecosphere* **2**(10), 117 (2011).

[32] Cortes Sanchez-Mata (de), M., & Tardio, J. *Mediterranean Wild Edible Plants Ethnobotany and Food Composition Tables*. (Springer, 2016).

[33] Groman-Yaroslavski, I., Weiss, E. & Nadel, D. Composite sickles and cereal harvesting methods at 23,000-years-Old Ohalo II, Israel. *PloS ONE* **11**, 11, e0167151 (2016). 10.1371/journal.pone.0167151 PMC5120854 27880839

[34] Van Zeist W, Bottema S (2009). A palynological study of the Acheulian site of Gesher Benot Ya’aqov, Israel. Veg. Hist. Archaeobot..

[35] Melamed Y (2016). The plant component of an Acheulian diet at Gesher Benot Ya‘aqov, Israel. PNAS.

[36] Willcox G (2005). The distribution, natural habitats and availability of wild cereals in relation to their domestication in the Near East: Multiple events, multiple centres. Veg. Hist. Archaeobot..

[37] Allaby RG, Fuller DQ, Brown TA (2008). The genetic expectations of a protracted model for the origins of domesticated crops. PNAS.

[38] Allaby RG (2015). Archaeogenomic insights into the adaptation of plants to the human environment pushing plant-hominin co-evolution back to the Pliocene. J. Hum. Evol..

[39] Fuller DQ, Asouti E, Purugganan MD (2012). Cultivation as slow evolutionary entanglement: Comparative data on rate and sequence of domestication. Veg. Hist. Archaeobot..

[40] Allaby RG (2017). Geographic mosaics and changing rates of cereal domestication. Philos. Trans. R. Soc. B..

[41] Zohary, D. & Hopf, M. *Domestication of Plants in the Old World*, 3rd edn. (Oxford University Press, 2000).

[42] Vaughan DA, Balazs E, Heslop-Harrison JS (2007). From crop domestication to super-domestication. Ann. Bot..

[43] Akçer-On S (2016). High Resolution Climatic Records of Western Anatolia for the Last 700 Ka: Acıgöl Lake Sedıments.

[44] Nakagawa T (1998). Dense-media separation as a more efficient pollen extraction method for use with organic sediment/deposit samples: Comparison with the conventional method. Boreas.

[45] Reille, M. *Pollen et spores d’Europe et d’Afrique du Nord*. (Laboratoire de Botanique Historique et de Palynologie, 1992).

[46] Reille, M. *Pollen et spores d’Europe et d’Afrique du Nord*, Supplément 1. (Laboratoire de Botanique Historique et de Palynologie, 1995).

[47] Reille, M. *Pollen et spores d’Europe et d’Afrique du Nord*, Supplément 2. (Laboratoire de Botanique Historique et de Palynologie, 1998).

[48] Beug H.J. *Leitfaden der Pollenbestimmung für Mitteleuropa und angrenzende Gebiete*. (Dr. Friedrich Pfeil, 2004).

[49] Cugny, C. *et al.* Modern and fossil non-pollen palynomorphs from the Basque mountains (western Pyrenees, France): The use of coprophilous fungi to reconstruct pastoral activity. *Veg. Hist. Archaeobot.* **19**, 391–408 (2010).

[50] Haas, J.N. (Ed.) Fresh insights into the palaeoecological and palaeoclimatological value of Quaternary non-pollen palynomorphs. *Veget. Hist. Archaeobot.* **19**, 389–560 (2010).

[51] Van Geel B, Aptroot A (2006). Fossil ascomycetes in Quaternary deposits. Nova Hedwigia.

[52] Djamali M, Cilleros K (2020). Statistically significant minimum pollen count in Quaternary pollen analysis; the case of pollen-rich lake sediments. Rev. Palaeobot. Palynol..

[53] Moore, P.D. *et al.**Pollen Analysis*. 2nd edn. (Blackwell, 1991).

[54] Bastin, B. Recherches sur les relations entre la végétation actuelle et le spectre pollinique récent dans la forêt de Soignes, Belgique. *Agricultura* XII (2ème série) 340–373 (1964).

[55] Leroi-Gourhan, A. Pollen grains of Gramineae and Cerealia from Shanidar and Zawi Chemi*,* in *The Domestication and Exploitation of Plants and Animals *143–148. (Gerald Duckworth & Co, 1969).

[56] Emery-Barbier A, Thiébault S (2005). Preliminary conclusions on the Late Glacial vegetation in south-west Anatolia the complementary nature of palynological and anthracological approaches. J. Archaeol. Sci..

[57] Faegri, K. & Iversen, J. *Textbook of Pollen Analysis*. 4th edn (revised by Faegri K., Kaland P.E., Krzywinski K.). (Wiley, 1989).

