# Author Correction: Continuous presence of proto-cereals in Anatolia since 2.3 Ma, and their possible co-evolution with large herbivores and hominins

**DOI:** 10.1038/s41598-022-06538-4

**Published:** 2022-02-08

**Authors:** Valérie Andrieu-Ponel, Pierre Rochette, François Demory, Hülya Alçiçek, Nicolas Boulbes, Didier Bourlès, Cahit Helvacı, Anne-Elisabeth Lebatard, Serdar Mayda, Henri Michaud, Anne-Marie Moigne, Sébastien Nomade, Mireille Perrin, Philippe Ponel, Claire Rambeau, Amélie Vialet, Belinda Gambin, Mehmet Cihat Alçiçek

**Affiliations:** 1 Institut Méditerranéen de Biodiversité et d’Ecologie Marine et Continentale (IMBE), Aix Marseille Univ, Avignon Université, CNRS, IRD, IMBE, Aix-en-Provence, France, Technopôle de l’Environnement Arbois-Méditerranée, BP 80, 13545 Aix-en-Provence Cedex 4, France; 2 Aix-Marseille University, CNRS, IRD, INRAE, UM 34 CEREGE, Technopôle de l’Environnement Arbois-Méditerranée, BP 80, 13545 Aix-en-Provence Cedex 4, France; 3 Department of Geology, Pamukkale University, 20070 Denizli, Turkey; 4 Laboratoire de Préhistoire, Muséum national d’Histoire naturelle, UMR 7194, UPVD, CERP, Avenue Léon Jean Grégory, 66720 Tautavel, France; 5 Department of Geology, Dokuz Eylül University, 35160 İzmir, Turkey; 6 Department of Biology, Ege University, 35100 İzmir, Turkey; 7 Conservatoire Botanique National Méditerranéen de Porquerolles, 34 Av. Gambetta, 83400 Hyères, France; 8 Laboratoire des Sciences du Climat et de l’Environnement (IPSL-CEA-CNRS UMR 8212-UVSQ), CEA Saclay, Site de L’orme Des Merisiers, Bât 714, 91198 Gif Sur Yvette, France; 9 LIVE, UMR7362, Université de Strasbourg, 3 rue de l’Argonne, 67000 Strasbourg, France; 10 Institute of Earth Systems, University of Malta, Msida, Malta

Correction to: *Scientific Reports* 10.1038/s41598-021-86423-8, published online 26 April 2021

The data access information included originally in the Article is not valid any more. The data has now been deposited in zenodo, and in the Data Availability section,

“All data generated or analysed during this study are included in this published article (and its Supplementary Information files) and are available on the e-site of EPD (European Pollen Database): http://www.europeanpollendatabase.net/data/downloads/image/ACIGOL3.zip.”

now reads:

“All data generated or analysed during this study are included in this published article (and its Supplementary Information files) and are available in zenodo at https://zenodo.org/record/5912616.”

The original Article has been corrected.

