# Supplementary material for: Continuous presence of proto-cereals in Anatolia since 2.3 Ma, and their possible co-evolution with large herbivores and hominins
Source: Sci Rep. 2021 Apr 26;11:8914. doi: 10.1038/s41598-021-86423-8 (PMC8076274; doi:10.1038/s41598-021-86423-8)
Supplement: Supplementary file 1 — Supplementary Information. [file 41598_2021_86423_MOESM1_ESM.docx]

**Continuous presence of proto-cereals in Anatolia since 2.3 Ma, and their possible co-evolution with large herbivores and hominins.**

Valérie Andrieu-Ponel^1^, Pierre Rochette^2^, François Demory^2^, Hülya Alçiçek^3^, Nicolas Boulbes^4^, Didier Bourlès^2^, Cahit Helvaci^5^ Anne-Elisabeth Lebatard^2^, Serdar Mayda^6^, Henri Michaud^7^, Anne-Marie Moigne^4^, Sébastien Nomade^8^, Mireille Perrin^2^, Philippe Ponel^1^, Claire Rambeau^9^, Amélie Vialet^4^, Belinda Gambin^10^ Mehmet Cihat Alçiçek^3^

1 - Institut Méditerranéen de Biodiversité et d'Ecologie marine et continentale (IMBE), Aix Marseille Univ, Avignon Université, CNRS, IRD, IMBE, Aix-en-Provence, France, Technopôle de l'Environnement Arbois-Méditerranée, BP 80, 13545 Aix-en-Provence Cedex 4, France. [valerie.andrieu@imbe.fr](mailto:valerie.andrieu@imbe.fr)

2 - Aix-Marseille University, CNRS, IRD, Coll. France, INRAE, UM 34 CEREGE, Technopôle de l'Environnement Arbois-Méditerranée, BP 80, 13545 Aix-en-Provence Cedex 4, France.

3 - Pamukkale University, Department of Geology, 20070, Denizli, Turkey.

4 - Laboratoire de Préhistoire, Muséum national d'Histoire naturelle, UMR 7194, UPVD, CERP, avenue Léon Jean Grégory, 66720 Tautavel, France.

5 - Dokuz Eylül University Department of Geology, 35160 İzmir, Turkey.

6 - Ege University, Department of Biology, 35100, İzmir, Turkey.

7 – Conservatoire botanique national méditerranéen de Porquerolles, 34 Av. Gambetta, 83400 Hyères, France.

8 - Laboratoire des Sciences du Climat et de L'Environnement (IPSL-CEA-CNRS 8212-UVSQ), CEA Saclay, Site de l’orme des Merisiers, Bât 714, 91198 Gif Sur Yvette, France.

9 - Université de Strasbourg, LIVE – UMR 7362, 3 rue de l'Argonne, 67000 Strasbourg, France.

10 - Institute of Earth Systems, University of Malta, Msida, Malta.

**Supplementary information**

**The sedimentary archive**

Lake Acıgöl (SW Anatolia, 37°49'N, 29°53'E, 836 m alt., Fig. 1) developed in a tectonic basin over a long-term period, evaluated to at least 3.4 Ma^1^. The lake belongs to a set of fluvio‐lacustrine deposits created in SW Anatolia by late Cenozoic extension processes. Lake level variations, as well as active subsidence due to a normal fault located on the basin’s southern margin^2^ controlled the sedimentary dynamics. Nowadays, this is a shallow endorheic lake (max depth: 1.63 m), fed by highly sulphated springs, and varying from 35 km^2^ to 100 km^2^ in area^3^. Sedimentological, geochemical, and mineralogical analyses were carried out on the 601 m long core 3 drilled by the ALKIM company^4,5^. Regular alternations between levels more or less rich in detrital fraction and in carbonates are observed. On the long term, there is a general shift from more carbonate-rich sediments (lower part) to more clay-rich deposits (upper part) at around 300 m depth^4,5^. High gypsum contents also seem to occur only in certain sections of the core. These can be related either to environmental conditions (higher evaporation at times of generally low lake levels), or to fluid circulations, possibly along tectonic breaches^4,5^. This first assessment showed that the lake evolved through time from deeper and perennial to shallower environments characterised by evaporation and precipitation processes (*e.g.* sodium sulphates, magnesium carbonates). More general studies demonstrated that the Acıgöl basin registered a constant interaction between tectonic processes, sedimentation, climate and chemistry since the end of the Miocene^6,4,5^. The present-day lake was formed through progressive narrowing and localised subsidence^4^.

Such long sedimentary records are rare on continents. The only other available long lacustrine sequences in Eurasia are found away from the major migratory routes followed by hominins (*e.g.* Lake Baikal, southern Russia^7^, and Lake Elgygytgyn, Russian Arctic^8^), or cover only shorter time periods, typically less than 1.5 Ma (Lake Ohrid, Albania^9^; Tenaghi Philippon, Greece^10^; Lake Van, Turkey^11^).

**Biogeography and vegetation**

From a biogeographic and bioclimatic point of view, the Acıgöl basin is in the Mediterranean area, with arid conditions that characterise the Anatolian intracontinental plateau^12,13^. The climate is typically Mediterranean (average annual temperature: 13.3°C), continental and arid (rainfall: 400 mm/yr), with minimum rainfall in summer and maximum rainfall in winter^13,3^. The current vegetation is characterised by a great taxonomic diversity, which results from the geographic position of Anatolia (a biogeographical crossroad), a long history (the region was mildly affected by Pleistocene glaciations), and from the diversity of altitudes and climates due to the complexity of relief around Acıgöl Lake. Lowland landscapes are dominated by steppes composed of shrubby plants^12^. Most of this steppe is now converted into cultivated or grazed grounds, and cereal fields have replaced the natural vegetation.

**Vegetation and climate history: state of art**

Whilst there are a significant number of studies devoted to the geodynamic setting and tectonic development of the area^14,15^, palaeoenvironmental and palaeoclimatic reconstructions by means of biological and geological analyses of sedimentary records from ancient lakes and wetlands in Anatolia are rare^16-22^. Due to poor preservation of the sporo-pollinic material in previous studies, landscape and climate reconstruction rely on low-resolution and imprecise records, giving only general indications on the flora and vegetation since the Miocene. From the Miocene to Pleistocene, the flora was first mega-mesothermic becoming arid-temperate in most of Anatolia when glacial-interglacial cycles of the Quaternary developed in the Northern Hemisphere^23^ except in some sites of N. central Anatolia^20^. The calcareous tufa of Sarıkavak (Acıgöl graben, SW Turkey) show a pollen record from Oxygen Isotope Stages (OIS) 11 to OIS 9, indicating a vegetation that was alternatively dominated by forests (Pinaceae mainly, Fagaceae, *Cedrus* and Oleaceae) and steppe-forests with Asteraceae and Chenopodiaceae^24^. The middle to late Pleistocene vegetation from OIS 6 to OIS 5 is recorded in the travertine of Kocabaş (Denizli, SW Turkey) and is characterised by open vegetation during the OIS 6 glaciation and by a diversified forest vegetation during the OIS 5 interglacial^25^. A discontinuous pollen record of the last eight climatic cycles is recorded in the Eskişehir Graben (Central Anatolia), showing a steppe-like vegetation cover, dominated by Asteraceae during glacials that alternate with coniferous forests dominated by *Pinus* during wet interglacial periods^26^.

1. Van den Hoek Ostende, L.W. *et al*. Ericek, a new Pliocene vertebrate locality in the Çameli Basin (southwestern Anatolia, Turkey). *Palaeobio Palaeoenv*. **95**, 305-320 (2015).

2. Alçiçek, H. Late Miocene non-marine sedimentation and formation of magnesites in the Acıgöl Basin, southwestern Anatolia, Turkey. *Sedimentary Geology* **219**, 115-135 (2009).

3. Garrett, D.E. *Sodium Sulphate: Handbook of Deposits, Processing, and Use*. Academic Press, 384 pp. (2001).

4. Helvacı, C. *et al*. Tectonosedimentary development and palaeoenvironmental changes in the Acıgöl shallow-perennial playa-lake basin, SW Anatolia, Turkey*. Turkish Journal of Earth Sciences* **22**, 173-190 (2013).

5. Demory, F. *et al*. Chronostratigraphy, depositional patterns and climatic imprints in Lake Acıgöl (SW Anatolia) during the Quaternary. *Quaternary Geochronology* **56**, 101038 (2020).

6. Alçiçek, M.C. *et al*. *Comparative researches of lacustrine deposits and paleosoils within fills of Acıgöl, Baklan and Dinar grabens deducing Pliocene and Quaternary climatic changes of SW Anatolia: A case study on interactions between climate and sedimentation*. Unpublished project report of TUBITAK (grant no: 105Y280), 302 pp. Ankara (2011).

7. Williams, D.F. *et al.* Lake Baikal record of continental climate response to orbital insolation during the past 5 million years. *Science* **278**, 1114-1117 (1997).

8. Melles, M. *et al.* 2.8 Million Years of Arctic Climate Change from Lake El’gygytgyn, NE Russia. *Science* **337**, 315-320 (2012).

9. Wagner, B. *et al*. Mediterranean winter rainfall in phase with African monsoons during the past 1.36 million years. *Nature* **573**, 256-260 (2019).

10. Pross, J. *et al.* The 1.35-Ma-long terrestrial climate archive of Tenaghi Philippon, northeastern Greece: Evolution, exploration and perspectives for future research. *Newsletters on Stratigraphy* **48**, 253-276 (2015).

11. Litt, T. & Anselmetti, F.S. Lake Van deep drilling project PALEOVAN. *Quaternary Science Reviews* **104**, 1-7 (2014).

12. Quézel P. & Barbero M. Carte de la végétation potentielle de la région méditerranéenne. Éditions du Centre National de la Recherche Scientifique (1985).

13. Akman, Y. & Ketenoǧlu, O. The climate and vegetation of Turkey. *Proceedings of the Royal Society of Edinburgh*, Section B: Biological Sciences **89**, 123-134 (1986).

14. Bozkurt, E. Neotectonics of Turkey, a synthesis. *Geodinamica Acta* **14**, 1-3, 3-30 (2001).

15. Ten Veen, J.H. *et al.* From palaeotectonics to neotectonics in the Neotethys realm. The importance of kinematic decoupling and inherited structural grain in SW Anatolia (Turkey). *Tectonophysics* **473**, 261-281 (2009).

16. Alçiçek, H. *et al.* Sedimentary facies, depositional environments and palaeogeographic evolution of the Neogene Denizli Basin of SW Anatolia, Turkey. *Sedimentary Geology* **202**, 596-637 (2007).

17. Altın, T.B. *et al.* N., Environmental and climatic changes during the Pleistocene–Holocene in the Bor Plain, Central Anatolia, Turkey. *Palaeogeography, Palaeoclimatology, Palaeoecology* **440**, 564-578 (2015).

18. Biltekin, D. *et al.* Anatolia: A long-time plant refuge area documented by pollen records over the last 23 million years. *Review of Palaeobotany and Palynology* **215**, 1-22 (2015).

19. Jiménez-Moreno, G. *et al.* Vegetation and climate changes during the late Pliocene and early Pleistocene in SW Anatolia, Turkey. *Quaternary Research* **84**, 448-456 (2015).

20. Kayseri-Özer, M.S. *et al.* Palaeoclimatic and palaeoenvironmental interpretations of the Late Oligocene, Late Miocene-Early Pliocene in the Çankiri-Çorum Basin. *Palaeogeography, Palaeoclimatology, Palaeoecology* **467**, 16-36 (2017).

21. Trifonov, V.G. *et al.* Pliocene Early Pleistocene history of the Euphrates valley applied to Late Cenozoic environment of the northern Arabian Plate and its surrounding, eastern Turkey. *Quaternary International* **493**, 137–165 (2018).

22. Rausch L. *et al.* An integrated reconstruction of the early Pleistocene palaeoenvironment of *Homo erectus* in the Denizli Basin (SW Turkey). *Geobios* **57**, 77-95 (2019).

23. Combourieu Nebout, N. & Vergnaud Grazzini, C. Late Pliocene northern hemisphere glaciations: the continental and marine responses in the central Mediterranean. *Quaternary Science Reviews* **10**, 319-334 (1991).

24. Tagliasacchi, E. & Kayseri-Özer, M.S. Palaeoclimate Changes, Acıgöl, Middle-Late Pleistocene, signals from Calcareous Tufa Pollen & Stable Isotope Records. *Alpine and Mediterranean Quaternary*, **31**, 161-164 (2018).

25. Toker, E. *et al.* Depositional system, pollen and palaeoclimatic interpretations of Middle to Late Pleistocene travertines Kocabaş, Denizli, south-west Turkey. *Sedimentology* **62**, 1360–1383 (2015).

26. Ocakoğlu, F. & Akkiraz, M.S. A Lower Pleistocene to Holocene terrestrial record from the Eskişehir Graben (Central Anatolia): Paleoclimatic and morphotectonic implications. *Quaternary International* **510**, 88-99 (2019).

27. Lebatard, A.E. *et al.* Dating the *Homo erectus* bearing travertine from Kocabaş (Denizli, Turkey) at least 1.1 Ma. *Earth and Planetary Science Letters* **390**, 8-18 (2014).

28. Cronquist, A. An integrated system of classification of flowering plants. New York: Columbia University Press (1981).

29. Couplan, F. La cuisine sauvage. Comment accommoder mille plantes oubliées. Encyclopédie des plantes comestibles de l'Europe, vol. 2. Equilibres Ed., Condé-sur-Noireau, 512 p. (1989).

30. Bonnier, G. & Douin, R. Flore complète illustrée en couleurs de France, Suisse et Belgique. Re-édition moderne, 1990. Belin, Paris. T. 1 + T.2 (Iconog.): XXXV + 924 p.; T.3 + T.4 (Texte): XXXI + 1401 p.; Index: XV + 191 p. (1911-1935).
