# Supplementary figures and images for: Continuous presence of proto-cereals in Anatolia since 2.3 Ma, and their possible co-evolution with large herbivores and hominins
Source: Sci Rep. 2021 Apr 26;11:8914. doi: 10.1038/s41598-021-86423-8 (PMC8076274; doi:10.1038/s41598-021-86423-8)

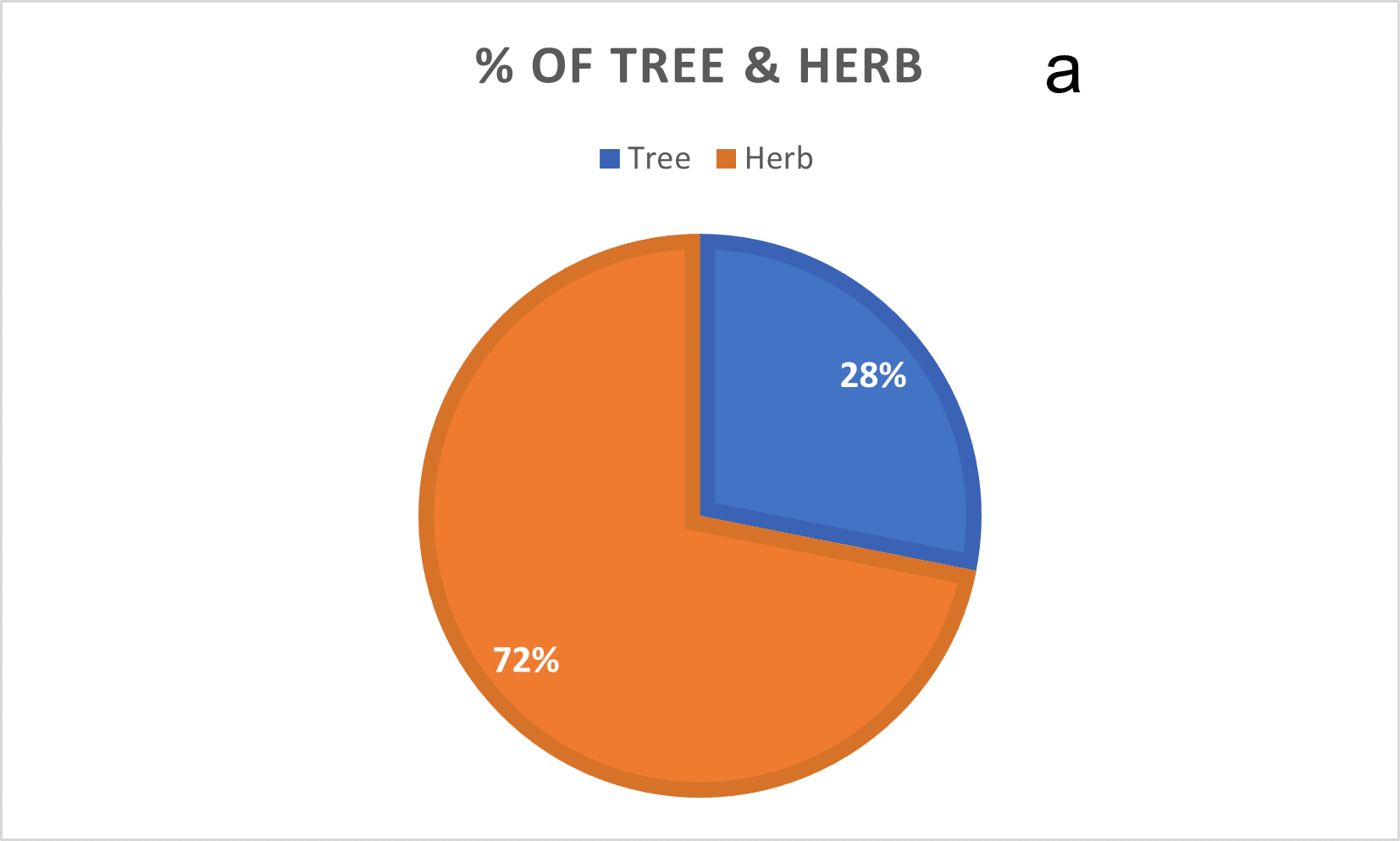


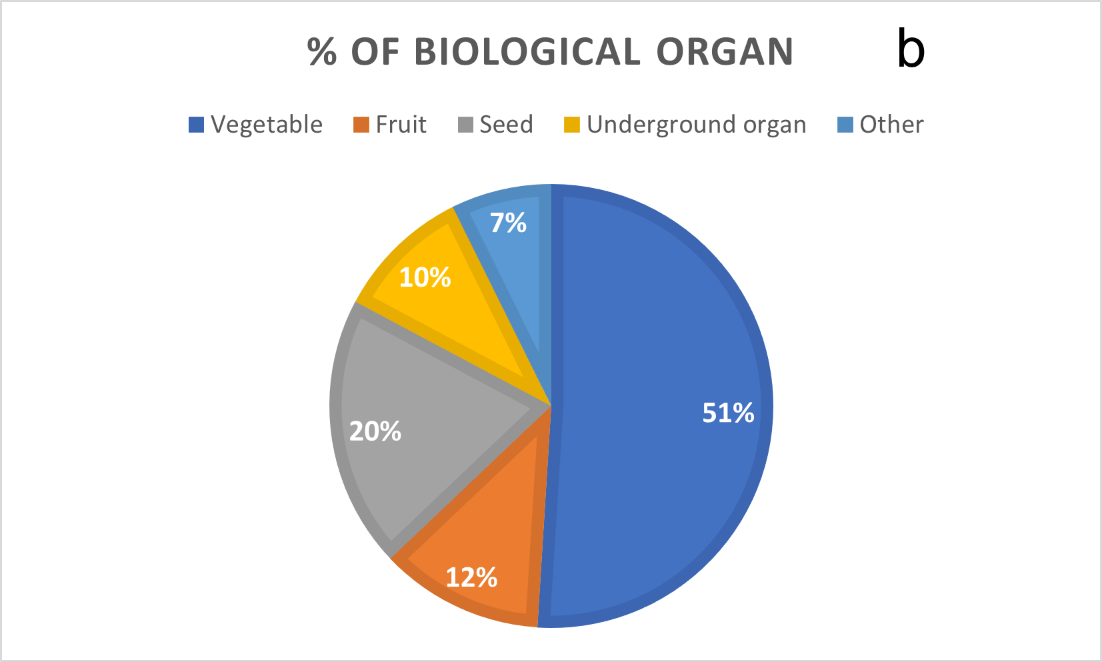

Supplement: Supplementary file 2 — Supplementary Figure 1. [file 41598_2021_86423_MOESM2_ESM.docx]
