# Supplementary material for: Continuous presence of proto-cereals in Anatolia since 2.3 Ma, and their possible co-evolution with large herbivores and hominins
Source: Sci Rep. 2021 Apr 26;11:8914. doi: 10.1038/s41598-021-86423-8 (PMC8076274; doi:10.1038/s41598-021-86423-8)
Supplement: Supplementary file 3 — Supplementary Table 1. [file 41598_2021_86423_MOESM3_ESM.docx]

| Age model (ka) | Wild Poaceae WP | Cerealia sum  C | WP+C | (C/WP+C)x100 |
| --- | --- | --- | --- | --- |
| 11.00 | 19.55 | 1.36 | 20.91 | 6.52 |
| 63.00 | 16.02 | 0.89 | 16.91 | 5.26 |
| 126.00 | 2.18 | 0.87 | 3.06 | 28.57 |
| 208.00 | 8.93 | 2.33 | 11.26 | 20.69 |
| 264.00 | 18.83 | 1.54 | 20.37 | 7.58 |
| 342.00 | 27.69 | 0.85 | 28.55 | 2.99 |
| 368.00 | 5.53 | 1.25 | 6.78 | 18.46 |
| 417.00 | 16.97 | 1.13 | 18.10 | 6.25 |
| 472.00 | 2.96 | 0.15 | 3.11 | 4.76 |
| 539.00 | 3.84 | 0.83 | 4.67 | 17.86 |
| 562.00 | 7.30 | 0.54 | 7.84 | 6.90 |
| 580.00 | 0.74 | 0.08 | 0.82 | 10.00 |
| 802.00 | 14.46 | 1.45 | 15.91 | 9.09 |
| 816.00 | 26.37 | 2.47 | 28.85 | 8.57 |
| 826.00 | 12.87 | 1.73 | 14.60 | 11.86 |
| 847.00 | 11.16 | 1.07 | 12.23 | 8.77 |
| 874.00 | 4.31 | 2.33 | 6.64 | 35.14 |
| 881.00 | 28.18 | 3.25 | 31.44 | 10.34 |
| 905.00 | 13.19 | 3.05 | 16.24 | 18.78 |
| 912.00 | 14.09 | 2.61 | 16.70 | 15.63 |
| 960.00 | 4.70 | 0.00 | 4.70 | 0.00 |
| 974.00 | 7.51 | 3.52 | 11.03 | 31.91 |
| 985.00 | 14.57 | 4.30 | 18.87 | 22.81 |
| 1009.00 | 7.76 | 0.55 | 8.31 | 6.67 |
| 1085.00 | 25.57 | 2.53 | 28.10 | 9.01 |
| 1143.00 | 12.14 | 1.46 | 13.59 | 10.71 |
| 1178.00 | 18.53 | 0.43 | 18.97 | 2.27 |
| 1218.00 | 5.52 | 1.66 | 7.18 | 23.08 |
| 1249.00 | 16.82 | 4.36 | 21.18 | 20.59 |
| 1297.00 | 19.91 | 0.22 | 20.13 | 1.10 |
| 1354.00 | 11.97 | 0.00 | 11.97 | 0.00 |
| 1385.00 | 11.75 | 1.81 | 13.55 | 13.33 |
| 1531.00 | 10.80 | 3.51 | 14.31 | 24.50 |
| 1553.00 | 6.96 | 0.87 | 7.83 | 11.11 |
| 1566.00 | 30.49 | 2.44 | 32.93 | 7.41 |
| 1597.00 | 18.80 | 1.06 | 19.86 | 5.33 |
| 1615.00 | 21.10 | 1.53 | 22.63 | 6.76 |
| 1615.00 | 6.80 | 0.30 | 7.10 | 4.17 |
| 1628.00 | 18.71 | 2.54 | 21.25 | 11.96 |
| 1659.00 | 4.31 | 1.23 | 5.54 | 22.22 |
| 1668.00 | 15.79 | 2.91 | 18.70 | 15.56 |
| 1690.00 | 29.27 | 6.19 | 35.46 | 17.46 |
| 1716.00 | 8.04 | 1.76 | 9.80 | 18.00 |
| 1738.00 | 1.86 | 0.59 | 2.44 | 24.00 |
| 1752.00 | 8.76 | 1.79 | 10.56 | 16.98 |
| 1778.00 | 7.19 | 2.16 | 9.35 | 23.08 |
| 1800.00 | 6.64 | 3.28 | 9.92 | 33.04 |
| 1811.00 | 8.22 | 0.55 | 8.77 | 6.25 |
| 1834.00 | 21.69 | 7.71 | 29.40 | 26.23 |
| 1874.00 | 8.41 | 1.09 | 9.50 | 11.43 |
| 1886.00 | 5.36 | 2.84 | 8.20 | 34.62 |
| 1908.00 | 4.52 | 2.41 | 6.93 | 34.78 |
| 1926.00 | 4.72 | 1.48 | 6.21 | 23.91 |
| 1934.00 | 17.41 | 1.71 | 19.11 | 8.93 |
| 1972.00 | 18.41 | 4.35 | 22.76 | 19.10 |
| 1991.00 | 22.07 | 5.43 | 27.50 | 19.75 |
| 2003.00 | 27.92 | 3.84 | 31.76 | 12.09 |
| 2022.00 | 1.61 | 1.34 | 2.95 | 45.45 |
| 2061.00 | 10.90 | 5.85 | 16.75 | 34.91 |
| 2088.00 | 23.81 | 3.61 | 27.42 | 13.16 |
| 2123.00 | 21.02 | 1.50 | 22.52 | 6.67 |
| 2142.00 | 13.35 | 7.12 | 20.47 | 34.78 |
| 2169.00 | 17.48 | 9.25 | 26.74 | 34.62 |
| 2193.00 | 16.72 | 5.69 | 22.41 | 25.37 |
| 2204.00 | 18.93 | 5.36 | 24.29 | 22.08 |
| 2239.00 | 25.21 | 3.84 | 29.04 | 13.21 |
| 2289.00 | 5.62 | 1.78 | 7.40 | 24.00 |
| 2305.00 | 12.09 | 6.87 | 18.96 | 36.23 |
| 2320.00 | 10.63 | 2.01 | 12.64 | 15.91 |
| 2339.00 | 8.82 | 5.00 | 13.82 | 36.17 |
| 2347.00 | 18.32 | 3.56 | 21.88 | 16.28 |
| 2355.00 | 20.54 | 6.36 | 26.89 | 23.64 |
| Sum (n=72) | 971.24 | 183.34 | 1154.59 | 1216.63 |
| Mean (n=72) | 13.49 | 2.55 | 16.04 | 16.90 |
| Mean 0-0.5 Ma n=9 | 13.19 | 1.15 | 14.34 | 11.33 |
| 0.5-1 Ma n=14 | 11.66 | 1.95 | 13.61 | 14.83 |
| 1-1.5 Ma n=9 | 14.44 | 1.45 | 15.89 | 9.64 |
| 1.5-2 Ma n=24 | 12.76 | 2.49 | 15.25 | 17.77 |
| 2-2.3 Ma n=16 | 15.81 | 4.56 | 20.37 | 24.66 |

Supplementary Table 1: Percentages of wild Poaceae (WP). Cerealia (C) and C/WP+C ratio from Acıgöl, core 3. The total number of pollen samples is 72. The age model is from Demory *et al*.,^5^.
