# Supplementary material for: Continuous presence of proto-cereals in Anatolia since 2.3 Ma, and their possible co-evolution with large herbivores and hominins
Source: Sci Rep. 2021 Apr 26;11:8914. doi: 10.1038/s41598-021-86423-8 (PMC8076274; doi:10.1038/s41598-021-86423-8)
Supplement: Supplementary file 4 — Supplementary Table 2. [file 41598_2021_86423_MOESM4_ESM.docx]

| Sample | Coordinates | Altitude (m) | Vegetation description | Floristic relevé |
| --- | --- | --- | --- | --- |
| 1 | N37°52'00.1" E29°51'25.6" | 839 | Lacustrine littoral.  Salted facies. Grassland with Chenopodiaceae and Salsolaceae | ***Aeluropus* sp**., ***Arthrocnemum fruticosum***, *Atriplex* sp., *HORDEUM* sp., *Hymenolobus procumbens*, *Lepidium perfoliatum*, *Limonium* sp., ***Puccinellia* sp**., *Salicornia* sp., *Salsola* sp., ***Sphenopus divaricatus*** |
| 2a | N37°52'05.0" E29°51'52.6" | 840 | Lacustrine littoral.  Salted facies. Grassland with Poaceae | *Achillea lycaonica*, ***Aegilops* sp**., *Astragalus* sp., *Atriplex* sp., *Carduus tenuiflorus*, *Convolvulus lineatus*, *Eleagnus angustifolia*, *Eruca sativa*, *Filago* sp., *Frankenia* sp., *Lepidium perfoliatum*, *Limonium* sp., *Malcolmia africana*, *Onopordon* sp., *Peganum harmala*, *Pinus brutia*, *Plantago lagopus*, ***Poa bulbosa***, *Podospermum laciniatum*, *Salsola* sp., *Tamarix* sp. |
| 3a | N37°51'49.3" E29°52'32.5" | 846 | Lacustrine littoral.  Salted facies. Grassland with Poaceae | *Astragalus* sp., *HORDEUM GENICULATUM*, *HORDEUM LEPORINUM*, ***Aeluropus littoralis***, *Convolvulus lineatus*, *Frankenia* sp., *Achillea lycaonica*, *Salsola* sp., *Salicornia* sp., ***Eremopyrum orientale***, ***Bromus* sp**., *Podospermum* *laciniatum*, ***Sphenopus* *divaricatus***, ***Parapholis* *pycnantha*** |
| 3b | N37°51'49.3" E29°52'32.5" | 846 | Lacustrine littoral.  Salted facies. Grassland with Chenopodiaceae and Salsolaceae. |  |
| 4 | N37°52'09.2" E29°52'59.7" | 843 | Lacustrine littoral.  Salted facies.  Grassland with Chenopodiaceae | ***Aeluropus* sp**., ***Arthrocnemum fruticosum***, *Atriplex* sp., *Bupleurum semicompositum*, *HORDEUM* sp., *Hymenolobus procumbens*, *Lepidium perfoliatum*, *Limonium* sp., ***Puccinellia* sp**., *Salicornia* sp., *Salsola* sp., ***Sphenopus* *divaricatus*** |
| 6 | N37°50'47.3" E29°58'36.3" | 843 | *Juncus* grassland on the lakeshore (freshwater facies) and, on the slope, evergreen oak forest with *Quercus calliprinos* (isolated individuals browsed by goats) | - On the lakeshore: ***Aeluropus* sp**., *Bellis perennis*, *Berula erecta*, *Carex distans*, *Carex divisa*, *Convolvulus arvensis*, *Convolvulus lineatus*, ***Cynodon dactylum***, *Eleocharis palustris*, *Juncus heldreichi*, *Lemna minor*, ***Phragmites australis***, *Plantago coronopus*, *Plantago major*, *Potentilla reptans*, *Ranunculus* sp., *Teucrium scorodonia*, *Trifolium fragiferum*, *Typha* sp. - On the slope: *Ajuga chamaepitys*, *Alyssum alyssoides*, *Amaranthus* sp., *Anthemis* sp., *Arabis verna*, *Arum dioscoridis*, *Calepina irregularis*, *Capsella bursapastoris*, *Convolvulus lineatus*, ***Echinaria capitate***, *Euphorbia* *helioscopia*, *Fumaria* sp., *Galium aparine*, *Lathyrus aphaca*, *Malva sylvestris*, *Medicago rigidula*, *Minuartia hamata*, *Nonnea echioides*, *Ornithogalum* sp., *Parietaria lusitanica*, ***Poa bulbosa***, *Quercus calliprinos*, *Rubia* sp., *Rumex pulcher* |
| 7a | N37°49'02.7" E29°55'37.1" | 840 | On the slopes,  just above the lacustrine littoral (freshwater facies), monospecific open grassland with *Verbascum,* and grazed evergreen oak forest with *Quercus calliprinos* | *Arum dracunculoides*, *Capsella bursapastori***s**, *Crepis sancta*, *Euphorbia rigida*, *Hypecoum imberbe*, *Juniperus excelsa*, *Quercus calliprinos*, ***Sclerochoa dura***, *Sisymbrium officinale*, *Verbascum* sp. |
| 7b | N37°49'02.7" E29°55'37.1" | 840 | Lacustrine littoral (freshwater facies), thermal spring. Grassland with *Juncus* sp., *Typha* sp., Phragmites | *Berula erecta*, *Galium aparine tenerum*, *Juncus heldreichi*, *Lythrum salicaria*, ***Phragmites australis***, *Plantago major*, ***Poa trivialis***, *Samolus valerandi*, *Schoenoplectus tabernaemontani*, *Typha dominguensis* |

Supplementary Table 2: Floristic relevé of 8 samples from the lakeshore of the Acıgöl lake and the surrounding slope. The relevé was carried out on 18 May 2017. Capitals: cereals, bold: wild Poaceae, underlined: Chenopodiaceae.
