# Supplementary material for: Continuous presence of proto-cereals in Anatolia since 2.3 Ma, and their possible co-evolution with large herbivores and hominins
Source: Sci Rep. 2021 Apr 26;11:8914. doi: 10.1038/s41598-021-86423-8 (PMC8076274; doi:10.1038/s41598-021-86423-8)
Supplement: Supplementary file 5 — Supplementary Table 3. [file 41598_2021_86423_MOESM5_ESM.docx]

|  | Samples | Chenopodiaceae sum | Wild Poaceae | Cereal  sum | Cerealia  40 µm | Cerealia  42.5 µm | Cerealia  45 µm | Cerealia  47.5 µm | Cerealia  52.5 µm | Cerealia  65 µm | *Secale* |
| --- | --- | --- | --- | --- | --- | --- | --- | --- | --- | --- | --- |
| Salted  facies | 1 | 57.00 | 8.80 | 0.12 | 0.12 | 0.00 | 0.00 | 0.00 | 0.00 | 0.00 | 0.00 |
|  | 2a | 43.44 | 10.97 | 0.97 | 0.00 | 0.32 | 0.43 | 0.11 | 0.11 | 0.00 | 0.00 |
|  | 3a | 29.62 | 10.79 | 0.37 | 0.00 | 0.00 | 0.00 | 0.00 | 0.00 | 0.00 | 0.37 |
|  | 3b | 52.44 | 6.72 | 0.00 | 0.00 | 0.00 | 0.00 | 0.00 | 0.00 | 0.00 | 0.00 |
|  | 4 | 21.57 | 7.02 | 0.33 | 0.00 | 0.00 | 0.00 | 0.00 | 0.17 | 0.00 | 0.17 |
| Freshwater facies | 6 | 1.61 | 0.54 | 0.00 | 0.00 | 0.00 | 0.00 | 0.00 | 0.00 | 0.00 | 0.00 |
|  | 7a | 0.95 | 0.82 | 0.00 | 0.00 | 0.00 | 0.00 | 0.00 | 0.00 | 0.00 | 0.00 |
|  | 7b | 0.84 | 2.53 | 0.21 | 0.00 | 0.00 | 0.00 | 0.00 | 0.00 | 0.21 | 0.00 |

Supplementary Table 3: Pollen analysis of the present pollen rain of the Acıgöl lake. Selected taxa. Values are in percentages calculated on the pollen sum of the pollen assemblages.
