# Supplementary material for: Continuous presence of proto-cereals in Anatolia since 2.3 Ma, and their possible co-evolution with large herbivores and hominins
Source: Sci Rep. 2021 Apr 26;11:8914. doi: 10.1038/s41598-021-86423-8 (PMC8076274; doi:10.1038/s41598-021-86423-8)
Supplement: Supplementary file 6 — Supplementary Table 4. [file 41598_2021_86423_MOESM6_ESM.docx]

|  | Pollen diameter (µm) | Number |
| --- | --- | --- |
| Wild Poaceae | 12.5 | 2 |
|  | 15 | 7 |
|  | 17.5 | 9 |
|  | 20 | 49 |
|  | 22.5 | 90 |
|  | 25 | 69 |
|  | 27.5 | 60 |
|  | 30 | 28 |
|  | 32.5 | 14 |
|  | 35 | 8 |
|  | 37.5 | 2 |
| Cereal  (C) | 40 | 2 |
|  | 42.5 | 4 |
|  | 45 | 6 |
|  | 47.5 | 1 |
|  | 50 | 1 |
|  | 52.5 | 1 |
|  | 55 | 1 |
|  | (C/Poaceae)x100 | 4.52 |

Supplementary Table 4: Wild Poaceae and cereal in the current pollen rain of Acıgöl. Numbers are in counting values. The C/Poaceae ratio is calculated on the sum of all Poaceae (wild Poaceae and cereal).
