# Supplementary material for: Continuous presence of proto-cereals in Anatolia since 2.3 Ma, and their possible co-evolution with large herbivores and hominins
Source: Sci Rep. 2021 Apr 26;11:8914. doi: 10.1038/s41598-021-86423-8 (PMC8076274; doi:10.1038/s41598-021-86423-8)
Supplement: Supplementary file 7 — Supplementary Table 5. [file 41598_2021_86423_MOESM7_ESM.docx]

| Taxa | Family | Edible organ | Tree | Herb | Vegetable | Fruit | Seed | Underground organ | Other |
| --- | --- | --- | --- | --- | --- | --- | --- | --- | --- |
| *Abies* sp. | Pinaceae | Inner bark | 1 |  |  |  |  |  | 1 |
| *Allium* sp. | Alliaceae | Bulb, leaf |  | 1 | 1 |  |  | 1 |  |
| *Ammi* t. | Apiaceae | Seed |  | 1 |  |  | 1 |  |  |
| Apiaceae | Apiaceae | Leaf, stem, root |  | 1 | 2 |  |  | 1 |  |
| *Arbutus* sp. | Ericaceae | Fruit | 1 |  |  | 1 |  |  |  |
| *Artemisia* sp. | Compositae | Leaf, shoot |  | 1 | 2 |  |  |  |  |
| *Asphodelus* sp. | Liliaceae | All |  | 1 | 1 | 1 | 1 | 1 | 1 |
| *Aster* t. | Compositae | Leaf, stem |  | 1 | 2 |  |  |  |  |
| *Atriplex* t. | Chenopodiaceae | All |  | 1 | 1 | 1 | 1 | 1 | 1 |
| *Betula* sp. | Betulaceae | Leaf, bark, sap | 1 |  | 1 |  |  |  | 2 |
| *Calendula* t. | Compositae | Leaf, flower |  | 1 | 2 |  |  |  |  |
| *Calluna* sp. | Ericaceae | Flower |  | 1 | 1 |  |  |  |  |
| *Calystegia* sp. | Convolvulaceae | Leaf |  | 1 | 1 |  |  |  |  |
| *Carduus* t. | Compositae | Leaf |  | 1 | 1 |  |  |  |  |
| *Carlina* t. | Compositae | Flower, stem, leaf |  | 1 | 3 |  |  |  |  |
| *Carya* sp. | Juglandaceae | Seed | 1 |  |  |  | 1 |  |  |
| *Castanea* sp. | Fagaceae | Fruit | 1 |  |  | 1 |  |  |  |
| *Celtis* sp. | Cannabaceae | Fruit, seed | 1 |  |  | 1 | 1 |  |  |
| *Centaurea collina* t. | Compositae | Flower |  | 1 | 1 |  |  |  |  |
| *Centaurea nigra* t. | Compositae | Flower |  | 1 | 1 |  |  |  |  |
| *Centranthus* t. | Valerianaceae | Leaf, flower |  | 1 | 2 |  |  |  |  |
| Cerealia 40 µm | Poaceae | Seed |  | 1 |  |  | 1 |  |  |
| Cerealia 42,5 µm | Poaceae | Seed |  | 2 |  |  | 2 |  |  |
| Cerealia 45 µm | Poaceae | Seed |  | 1 |  |  | 1 |  |  |
| Cerealia 47,5 µm | Poaceae | Seed |  | 1 |  |  | 1 |  |  |
| Cerealia 50 µm | Poaceae | Seed |  | 1 |  |  | 1 |  |  |
| Cerealia 52,5 µm | Poaceae | Seed |  | 1 |  |  | 1 |  |  |
| Cerealia 55 µm | Poaceae | Seed |  | 1 |  |  | 1 |  |  |
| Cerealia 57,5 µm | Poaceae | Seed |  | 1 |  |  | 1 |  |  |
| Cerealia 60 µm | Poaceae | Seed |  | 1 |  |  | 1 |  |  |
| Cerealia 62,5 µm | Poaceae | Seed |  | 1 |  |  | 1 |  |  |
| Cerealia 75 µm | Poaceae | Seed |  | 1 |  |  | 1 |  |  |
| Chenopodiaceae | Chenopodiaceae | Leaf |  | 1 | 1 |  |  |  |  |
| Cichorioideae | Cichorioideae | Flower, leaf, stem |  | 1 | 3 |  |  |  |  |
| *Cirsium* sp. | Compositae | Flower, leaf, stem |  | 1 | 3 |  |  |  |  |
| *Corylus* sp. | Betulaceae | Seed | 1 |  |  |  | 1 |  |  |
| Cyperaceae | Cyperaceae | Root |  | 1 |  |  |  | 1 |  |
| *Cyperus* sp. | Cyperaceae | Root |  | 1 |  |  |  | 1 |  |
| *Ephedra distachya* t. | Ephedraceae | Fruit |  | 1 |  | 1 |  |  |  |
| *Ephedra fragilis* t. | Ephedraceae | Fruit |  | 1 |  | 1 |  |  |  |
| *Erica arborea* t. | Ericaceae | Flower | 1 |  | 1 |  |  |  |  |
| Ericaceae | Ericaceae | Flower |  | 1 | 1 |  |  |  |  |
| *Erodium* sp. | Geraniaceae | Leaf |  | 1 | 1 |  |  |  |  |
| *Fagopyrum* sp. | Polygonaceae | Seed |  | 1 |  |  | 1 |  |  |
| *Fagus* sp. | Fagaceae | Seed | 1 |  |  |  | 1 |  |  |
| *Fraxinus* sp. | Oleaceae | Leaf | 1 |  | 1 |  |  |  |  |
| *Galium* sp. | Rubiaceae | Leaf |  | 1 | 1 |  |  |  |  |
| *Geranium* sp. | Geraniceae | Leaf |  | 1 | 1 |  |  |  |  |
| *Hippophae* sp. | Eleagnaceae | Fruit | 1 |  |  | 1 |  |  |  |
| *Juglans* sp. | Juglandaceae | Seed | 1 |  |  |  | 1 |  |  |
| *Juniperus* sp. | Cupressaceae | Fruit | 1 |  |  | 1 |  |  |  |
| Lamiaceae | Lamiaceae | All |  | 1 | 1 | 1 | 1 | 1 | 1 |
| *Linum* sp. | Linaceae | Seed |  | 1 |  |  | 1 |  |  |
| *Liquidambar* sp. | Altingiaceae | Sap | 1 |  |  |  |  |  | 1 |
| *Lotus* t. | Fabaceae | Seed |  | 1 |  |  | 1 |  |  |
| *Malva* sp. | Malvaceae | Seed, leaf, stem |  | 1 | 2 |  | 1 |  |  |
| *Mentha* t. | Lamiaceae | Leaf |  | 1 | 1 |  |  |  |  |
| *Montia* sp | Portulacaceae | Leaf |  | 1 | 1 |  |  |  |  |
| *Nymphaea* sp | Nympheaceae | Rhizome |  | 1 |  |  |  | 1 |  |
| *Olea* sp | Oleaceae | Fruit | 1 |  |  | 1 |  |  |  |
| *Onopordon* t. | Compositae | Flower, stem |  | 1 | 2 |  |  |  |  |
| *Papaver* t. | Papaveraceae | All |  | 1 | 1 | 1 | 1 | 1 | 1 |
| *Picea* sp. | Pinaceae | Bud | 1 |  | 1 |  |  |  |  |
| *Pimpinella* t. | Apiaceae | Seed |  | 1 |  |  | 1 |  |  |
| *Pinus sylvestris* t. | Pinaceae | Seed, young shoot | 1 |  | 1 |  | 1 |  |  |
| *Pinus* (Mediterranean) | Pinaceae | Seed, young shoot | 1 |  | 1 |  |  |  |  |
| *Pistacia* sp. | Anacardiaceae | Fruit, resin | 1 |  |  | 1 |  |  | 1 |
| *Plantago coronopus* t. | Plantaginaceae | Young leaf |  | 1 | 1 |  |  |  |  |
| *Plantago lanceolata* t. | Plantaginaceae | Young leaf |  | 1 | 1 |  |  |  |  |
| *Plantago major-media* t. | Plantaginaceae | Young leaf |  | 1 | 1 |  |  |  |  |
| Poaceae | Poaceae | Young shoot, leaf |  | 1 | 2 |  |  |  |  |
| *Populus* sp. | Salicaceae | Sap, young shoot, bud, bark | 1 |  | 2 |  |  |  | 1 |
| *Potamogeton* sp. | Scrofulariaceae | Rhizome |  | 1 |  |  |  | 1 |  |
| *Potentilla* t. | Rosaceae | All |  | 1 | 1 | 1 | 1 | 1 | 1 |
| *Prunella* t. | Lamiaceae | Young leaf |  | 1 | 1 |  |  |  |  |
| *Prunus* t. | Rosaceae | Fruit | 1 |  |  | 1 |  |  |  |
| *Pteridium aquilinum* | Dennstaedtiaceae | Rhizome, young shoot |  | 1 | 1 |  |  | 1 |  |
| *Quercus* (deciduous) | Fagaceae | Fruit | 1 |  |  | 1 |  |  |  |
| *Quercus calliprinos* t. | Fagaceae | Fruit | 1 |  |  | 1 |  |  |  |
| *Quercus cerris* t. | Fagaceae | Fruit | 1 |  |  | 1 |  |  |  |
| *Reseda* sp. | Resedaceae | Leaf, young shoot |  | 1 | 2 |  |  |  |  |
| *Rumex* sp. | Polygonaceae | Leaf, stem |  | 1 | 2 |  |  |  |  |
| *Salix* sp. | Salicaceae | Leaf, young shoot | 1 |  | 2 |  |  |  |  |
| *Sanguisorba minor* t. | Rosaceae | Leaf |  | 1 | 1 |  |  |  |  |
| *Secale* sp. | Poaceae | Seed |  | 1 |  |  | 1 |  |  |
| *Sedum* t. | Crassulaceae | Flower, young shoot |  | 1 | 2 |  |  |  |  |
| *Silene* t. | Caryophyllaceae | Leaf |  | 1 | 1 |  |  |  |  |
| *Sinapis* t. | Brassicaceae | Seed, leaf |  | 1 | 2 |  |  |  |  |
| *Sparganium-Typha* | Sparganiaceae | Root |  | 1 |  |  |  | 1 |  |
| *Stachys* t. | Lamiaceaea | Young shoot, leaf, flower |  | 1 | 3 |  |  |  |  |
| *Stellaria* t. | Caryophyllaceae | Young shoot, leaf |  | 1 | 2 |  |  |  |  |
| *Tilia* sp. | Tiliaceae | Young leaf, Flower | 1 |  | 2 |  |  |  |  |
| *Typha* sp. | Typhaceae | Shoot, rhizome |  | 1 |  |  |  | 2 |  |
| *Ulmus* sp. | Ulmaceae | Leaf | 1 |  | 1 |  |  |  |  |
| *Valeriana* sp. | Valerianaceae | Leaf |  | 1 | 1 |  |  |  |  |
| Sum of tree & herb | | | 27 | 69 |  |  |  |  |  |
| % of tree & herb | | | 28.1 | 71.9 |  |  |  |  |  |
| Sum of biological organ | | | | | 77 | 18 | 30 | 15 | 11 |
| % of biological organ | | | | | 51 | 11.9 | 19.9 | 9.9 | 7.3 |

Supplementary Table 5: Table of potentially edible plants identified in the pollen assemblages of Acıgöl. The systematic is from Cronquist^28^. Edible organs of the plants are from Couplan^29^ and Bonnier and Douin^30^.
