# Supplementary material for: Continuous presence of proto-cereals in Anatolia since 2.3 Ma, and their possible co-evolution with large herbivores and hominins
Source: Sci Rep. 2021 Apr 26;11:8914. doi: 10.1038/s41598-021-86423-8 (PMC8076274; doi:10.1038/s41598-021-86423-8)
Supplement: Supplementary file 8 — Supplementary Table 6. [file 41598_2021_86423_MOESM8_ESM.docx]

| Magnetic boundary | Depth (m) | Age (ka) |
| --- | --- | --- |
| Bruhnes-Matuyama | 210 | 781 |
| Top Jaramillo | 270 | 988 |
| Bottom Jaramillo | 290 | 1072 |
| Top Olduvai | 450 | 1778 |
| Bottom Olduvai | 495 | 1945 |

Supplementary Table 6. Magnetostratigraphy temporal tie points) of the series of Acıgöl, core 3
